# Supplementary material for: Efficiency of In-Store Interventions to Impact Customers to Purchase Healthier Food and Beverage Products in Real-Life Grocery Stores: A Systematic Review and Meta-Analysis
Source: Foods. 2021 Apr 22;10(5):922. doi: 10.3390/foods10050922 (PMC8146080; doi:10.3390/foods10050922)
Supplement: Supplementary file 1 [file foods-10-00922-s001.zip › foods-1184613-supplementary.pdf]

Supplementary material

Table S1. Application of the SPICE framework for search word selection

Setting	supermarket*; grocer*
Population	Include all populations
Intervention	nudg*; choice architect*; intervention*; behav*; behavioral economics; place*; product*; promot*; defaults; prim*; prompt*; option*; newsletter*; taste testing; flyers portion*; package*; size*; pric*; swaps; poster; label*; messag*; order; distance; option*;stock
Comparator	experiment*; field; random*; trail;
Evaluation	Choice; consumption; change; improve*; sale* eat; purchas*; health*; unhealth*; nutrition behave*

Comment: Specific search terms were developed according to the SPICE (Setting, Population; Intervention, Comparison, Evlauation framework (Booth 2006).

Table S2. Search words

TITLE-ABS-KEY (“super market*” OR supermarket* OR grocer*) AND
TITLE-ABS-KEY (eating OR purchas* OR consum* OR “diet* healthy” OR “healthy diet*” OR “diet* unhealthy” OR “unhealthy diet*” OR “health* behav*” OR “nutrition behav*”) AND
TITLE-ABS-KEY (nudg* OR marketing OR advertising OR “choice architect*” OR placement* OR product* OR promot* OR “health* default*” OR prim* OR prompt* OR newsletter* OR “taste testing” OR flyers OR portion* OR package* OR size* OR pric* OR swaps OR poster OR label* OR messag* OR order OR distance OR option* OR stock)

Limit
Journal Articles

Table S3. Search Strategies and yields

Database	Date	Yield
Cochrane Libray	24 April 2020	281 + 3 reviews
EconLit	24 April 2020	678
Medeline (Ovid)	24 April 2020	117
PsychInfo (Ovid)	24 April 2020	108
Scopus	24 April 2020	1193
Web of Science	24 April 2020	1875

Table S4. Excluded articles and reason for exclusion

Nr.	Source	Reference	Reason for exclusion
1	Database search	(Adam & Jensen, 2016)	Study design: Review article
2	Cadario and Chardon, 2020	(Adams, Pelletier, Zive, & Sallis, 2005)	Setting: Not in real grocery store.
3	Hersey et al. (2013)	(Guiding Stars Licensing Company, 2010a)	Publications: Articles published in non-peer reviewed journals
4	Database search	(Afshin et al., 2015)	Study design: Review article
5	Database search	(Ahmed, 2008)	Outcome: Interventions targeting sales of product that could not be categorized as healthy or unhealthy
6	Glanz, Bader & Iyer. (2012)	(Ailawadi, 2001)	Study design: Not experimental study design
7	Database search	(Akgungor, Groppel-Klein, Koenigstorfer, Gulcan, & Kustepeli, 2016)	Study design: Study did not fit study design criteria
8	Database search	(Alamsyah, Othman, & Mohammed, 2020)	Study design: Not experimental study design
9	Database search	(Almenar, Catala, Hernandez-Munoz, & Gavara, 2009)	Outcome: Interventions targeting sales of product that could not be categorized as healthy or unhealthy
10	Database search	(R. An, 2013)	Setting: Not in real grocery store.
11	Database search	(R. An & Sturm, 2017)	Study design: Not experimental study design
12	Epstein et al. (2012) + Liberato, Bailie & Brimblecombe. (2014) + Afshin et al. (2017)	(J. V. Anderson et al., 2001)	Setting: Not in real grocery store.
13	Database search	(E. T. Anderson & Simester, 2001)	Outcome: Interventions targeting sales of product that could not be categorized as healthy or unhealthy
14	Database search	(E. T. Anderson & Simester, 2004)	Outcome: Interventions targeting sales of product that could not be categorized as healthy or unhealthy
15	Database search	(Andorfer & Liebe, 2015)	Outcome: Interventions targeting sales of product that could not be categorized as healthy or unhealthy
16	Database search	(Andreis & Milan, 2016)	Outcome: Outcome not real change in consumption or purchase behavior
17	Hersey et al. (2013)	(Andrews, Burton, & Kees, 2011)	Outcome: Interventions targeting sales of product that could not be categorized as healthy or unhealthy
18	Glanz, Bader & Iyer. (2012)	(Andreyeva, Long, & Brownell, 2010)	Study design: Review article
19	Adam (2016)	(Andreyeva, Luedicke, Middleton, Long, & Schwartz, 2012)	Intervention: Not in-store intervention meeting criteria
20	Database search	(Ang, Agrawal, & Finkelstein, 2019)	Outcome: Interventions targeting sales of product that could not be categorized as healthy or unhealthy
21	Database search	(Anic & Vouk, 2005)	Outcome: Interventions targeting sales of product that could not be categorized as healthy or unhealthy
22	Database search	(Ankamah-Yeboah, Nielsen, & Nielsen, 2016)	Outcome: Interventions targeting sales of product that could not be categorized as healthy or unhealthy

23	Afskin et al. (2017)	(Anliker, Winne, & Drake, 1992)	Setting: Not in real grocery store.
24	Cadario and Chardon, 2020	(Anzman-Frasca et al., 2018)	Setting: Not in real grocery store.
25	Database search	(Arce, 2019)	Outcome: Outcome not real change in consumption or purchase behavior
26	Database search	(Arce-Urriza, Cebollada, & Tarira, 2017)	Outcome: Interventions targeting sales of product that could not be categorized as healthy or unhealthy
27	Database search	(Aschemann-Witzel & Aagaard, 2014)	Study design: Not experimental study design
28	Database search	(Asenslo & Montero, 2008)	Study design: Not experimental study design
29	Database search	(Astuty, 2019)	Study design: Not experimental study design
30	Cadario and Chardon, 2020	(Auchincloss et al., 2013)	Setting: Not in real grocery store.
31	Database search	(Azeem, Baker, Villano, Mounter, & Griffith, 2019)	Outcome: Interventions targeting sales of product that could not be categorized as healthy or unhealthy
32	Database search	(Bailey, Shaw, Alexander, & Nell, 2010)	Outcome: Interventions targeting sales of product that could not be categorized as healthy or unhealthy
33	Adam (2016)	(Bains et al., 2014)	Population: Population enrolled in study at health centers or other settings not directly related to grocery store setting.
34	Database search	(Bakini Driss, Ben Lallouna Hafsia, & Zghal, 2008)	Publications: Non-English Language publications
35	Hersey et al. (2013)	(Balcombe, Fraser, & Di Falco, 2010)	Publications: Articles published in non-peer reviewed journals
36	Database search	(K. Ball et al., 2011)	Study design: Not experimental study design
37	Database search	(K. Ball et al., 2013)	Study design: Not experimental study design
38	Database search + Hartman et al. (2018)	(Kylie Ball et al., 2016)	Setting: Not in real grocery store.
39	Cameron (2016)	(Bangia & Palmer-Keenan, 2014)	Outcome: Outcome not real change in consumption or purchase behavior
40	Database search	(Barat & Paswan, 2005)	Outcome: Interventions targeting sales of product that could not be categorized as healthy or unhealthy
41	Cadario and Chardon, 2020	(Bartholomew & Jowers, 2006)	Setting: Not in real grocery store.
42	Cadario and Chardon, 2020	(Baskin et al., 2016)	Setting: Not in real grocery store.
43	Database search	(Batte, Hooker, Haab, & Beaverson, 2007)	Outcome: Interventions targeting sales of product that could not be categorized as healthy or unhealthy
44	Database search	(Becchetti, Salustri, & Scaramozzino, 2019)	Setting: Not in real grocery store.
45	Glanz, Bader & Iyer. (2012)	(Bell, Corsten, & Knox, 2011)	Intervention: Not in-store intervention meeting criteria
46	Database search	(Bercik, Horska, Wang, & Chen, 2016)	Study design: Not experimental study design
47	Database search	(Bernales-Korins, Ang, Khan, & Geliebter, 2017)	Duplicates: Duplicate publications with Geliebter et al., 2013 included in review

48	Database search	(Berning, Chouinard, Manning, McCluskey, & Sprott, 2010)	Outcome: Interventions targeting sales of product that could not be categorized as healthy or unhealthy
49	Cameron (2016)	(Berning, Chouinard, & McCluskey, 2011)	Outcome: Outcome not real change in consumption or purchase behavior
50	Database search	(Berning, 2014)	Study design: Not experimental study design
51	Glanz, Bader & Iyer. (2012)	(Bezawada, Balachander, Kannan, & Shankar, 2009)	Study design: Not experimental study design
52	Database search	(Bialkova, Grunert, & van Trijp, 2020)	Study design: Not experimental study design
53	Adam (2016) + Afskin et al. (2017)	(Bihan et al., 2012)	Intervention: Not in-store intervention meeting criteria
54	Database search	(Biswas, Szocs, Chacko, & Wansink, 2017)	Intervention: Not in-store intervention meeting criteria
55	Database search	(Biswas & Szocs, 2019)	Study design: Not experimental study design
56	Glanz, Bader & Iyer. (2012)	(Black & Macinko, 2008)	Study design: Not experimental study design
57	Database search	(Blanck, Thompson, Nebeling, & Yaroch, 2011)	Setting: Not in real grocery store.
58	Adam (2016) + Glanz, Bader & Iyer. (2012) + Afskin et al. (2017) + Database search	(Blakely et al., 2011)	Outcome: Outcome not real change in consumption or purchase behavior
59	Glanz, Bader & Iyer. (2012)	(Blattberg, Briesch, & Fox, 1995)	Setting: Not in real grocery store.
60	Epstein et al. (2012) + Afskin et al. (2017)	(Block, Chandra, McManus, & Willett, 2010)	Setting: Not in real grocery store.
61	Database search	(Blomquist, Bartolino, & Waldo, 2015)	Outcome: Interventions targeting sales of product that could not be categorized as healthy or unhealthy
62	Glanz, Bader & Iyer. (2012	(Bodor, Rice, Farley, Swalm, & Rose, 2010)	Intervention: Not in-store intervention meeting criteria
63	Database search + Epstein et al. (2012)	(Bodor, Ulmer, Futrell Dunaway, Farley, & Rose, 2010)	Study design: Not experimental study design
64	Cadario and Chardon, 2020	(Bollinger, Leslie, & Sorensen, 2011)	Setting: Not in real grocery store.
65	Database search	(Bolton & Shankar, 2003)	Study design: Not experimental study design
66	Escaron, Meinen, Nitzke & Martines-Donete. (2013)	(Booth-Butterfield & Reger, 2004)	Setting: Not in real grocery store.
67	Hersey et al. (2013)	(Borgmeier & Westenhoefer, 2009)	Outcome: Outcome not real change in consumption or purchase behavior
68	Database search	(Borle, Boatwright, Kadane, Nunes, & Shmueli, 2005)	Study design: Not experimental study design
69	Database search	(Bower & Turner, 2001)	Outcome: Interventions targeting sales of product that could not be categorized as healthy or unhealthy
70	Database search	(Breugelmans & Campo, 2011)	Outcome: Interventions targeting sales of product that could not be categorized as healthy or unhealthy
71	Database search	(R. A. Briesch, Dillon, & Fox, 2013)	Outcome: Interventions targeting sales of product that could not be categorized as healthy or unhealthy

72	Database search + Glanz, Bader & Iyer. (2012)	(Richard A Briesch, Chintagunta, & Fox, 2009)	Outcome: Outcome not real change in consumption or purchase behavior
73	Cadario and Chardon, 2020	(Brissette, Lowenfels, Noble, & Spicer, 2013)	Setting: Not in real grocery store.
74	Glanz, Bader & Iyer. (2012)	(Broniarczyk, Hoyer, & McAlister, 1998)	Setting: Not in real grocery store.
75	Glanz, Bader & Iyer. (2012)	(Bronnenberg, Mela, & Boulding, 2006)	Setting: Not in real grocery store.
76	Afskin et al. (2017)	(Brown & Tammineni, 2009)	Setting: Not in real grocery store.
77	Database search	(Brownbill, Miller, & Braunack-Mayer, 2018)	Study design: Not experimental study design
78	Hartman et al. (2018)	(Budd et al., 2017)	Setting: Not in real grocery store.
79	Cadario and Chardon, 2020	(Buscher, Martin, & Crocker, 2001)	Setting: Not in real grocery store.
80	Database search	(Bustillos, Sharkey, Anding, & McIntosh, 2009)	Study design: Not experimental study design
81	Database search	(Butler & Vossler, 2018)	Setting: Not in real grocery store.
82	Adam (2016)	(Caldwell, Kobayashi, DuBow, & Wytinck, 2009)	Setting: Not in real grocery store.
83	Glanz, Bader & Iyer. (2012)	(Campo, Gijsbrechts, Goossens, & Verhetsel, 2000)	Outcome: Outcome not real change in consumption or purchase behavior
84	Database search	(Carroll, Bernard, & Pesek Jr, 2013)	Outcome: Interventions targeting sales of product that could not be categorized as healthy or unhealthy
85	Database search	(Carroll, Samek, & Zepeda, 2018)	Setting: Not in real grocery store.
86	Glanz, Bader & Iyer. (2012)	(Chandon & Wansink, 2002)	Intervention: Not in-store intervention meeting criteria
87	Database search + Glanz, Bader & Iyer. (2012)	(Chandon, Hutchinson, Bradlow, & Young, 2009)	Study design: Not experimental study design
88	Glanz, Bader & Iyer. (2012)	(Cheadle et al., 1991)	Study design: Not experimental study design
89	Glanz, Bader & Iyer. (2012)	(Cheadle et al., 1993)	Study design: Not experimental study design
90	Glanz, Bader & Iyer. (2012)	(Cheadle et al., 1995)	Population: Population enrolled in study at health centers or other settings not directly related to grocery store setting.
91	Database search	(Chintakayala, Young, Barkemeyer, & Morris, 2018)	Outcome: Interventions targeting sales of product that could not be categorized as healthy or unhealthy
92	Database search	(J. Chu, Chintagunta, & Cebollada, 2008)	Outcome: Interventions targeting sales of product that could not be categorized as healthy or unhealthy
93	Cadario and Chardon, 2020	(Y. H. Chu, Frongillo, Jones, & Kaye, 2009)	Setting: Not in real grocery store.
94	Epstein et al. (2012)	(Cinciripini, 1984)	Setting: Not in real grocery store.
95	Database search	(Clement, Aastrup, & Charlotte Forsberg, 2015)	Study design: Not experimental study design
96	Glanz, Bader & Iyer. (2012)	(Do Vale, Pieters, & Zeelenberg, 2008)	Setting: Not in real grocery store.
97	Database search	(Coffino, Udo, & Hormes, 2020)	Study design: Study did not fit study design criteria

98	Database search	(Cohen, Collins, Hunter, Ghosh-Dastidar, & Dubowitz, 2015)	Study design: Not experimental study design
99	Cadario and Chardon, 2020	(J. F. W. Cohen et al., 2015)	Setting: Not in real grocery store.
100	Database search	(Cohen & Babey, 2012)	Study design: Not experimental study design
101	Database search	(Cohen et al., 2018)	Study design: Not experimental study design
102	Escaron, Meinen, Nitzke & Martines-Donete. (2013) + Adam (2016) + Database search	(Colapinto & Malaviarachchi, 2009)	Outcome: Outcome not real change in consumption or purchase behavior
103	Database search + Cameron (2016)	(Cotugna & Vickery, 1992)	Outcome: Interventions targeting sales of product that could not be categorized as healthy or unhealthy
104	Database search	(Coucke, Vermeir, Slabbinck, & Van Kerckhove, 2019)	Outcome: Interventions targeting sales of product that could not be categorized as healthy or unhealthy
105	Escaron, Meinen, Nitzke & Martines-Donete. (2013)	(Crawford & Kalina, 1993)	Outcome: Outcome not real change in consumption or purchase behavior
106	Cadario and Chardon, 2020	(R. A. Crockett, Jebb, Hankins, & Marteau, 2014)	Setting: Not in real grocery store.
107	Database search	(Rachel A. Crockett et al., 2018)	Study design: Review article
108	Database search	(Cucchiara, Kwon, & Ha, 2015)	Outcome: Interventions targeting sales of product that could not be categorized as healthy or unhealthy
109	Glanz, Bader & Iyer. (2012)	(Cui, Raju, & Zhang, 2008)	Study design: Not experimental study design
110	Adam (2016)	(Cummins & Macintyre, 2006)	Study design: Not experimental study design
111	Cameron (2016)	(Curhan, 1974)	Study design: Not experimental study design
112	Adam (2016) + Escaron, Meinen, Nitzke & Martines-Donete. (2013	(Curran et al., 2005)	Study design: Not experimental study design
113	Database search	(Daliani, Stott, Fiore, & Cottin, 2019)	Study design: Not experimental study design
114	Adam (2016)	(Dannefer, Williams, Baronberg, & Silver, 2012)	Outcome: Outcome not real change in consumption or purchase behavior
115	Database search	(Dawson, 2013)	Study design: Not experimental study design
116	Cadario and Chardon, 2020	(Dayan & Bar-Hillel, 2011)	Setting: Not in real grocery store.
117	Cadario & Chardon, 2020	(De Bondt, Van Kerckhove, & Geuens, 2017)	Setting: Not in real grocery store.
118	Database search + Cadario & Chardon, 2020 + Cameron (2016)	(De Wijk et al., 2016)	Study design: Study did not fit study design criteria
119	Database search	(Degeratu, Rangaswamy, & Wu, 2000)	Outcome: Interventions targeting sales of product that could not be categorized as healthy or unhealthy
120	Database search	(Demarque, Charalambides, Hilton, & Waroquier, 2015)	Outcome: Interventions targeting sales of product that could not be categorized as healthy or unhealthy
121	Glanz, Bader & Iyer. (2012)	(K. K. Desai & Ratneshwar, 2003)	Outcome: Outcome not real change in consumption or purchase behavior

122	Glanz, Bader & Iyer. (2012)	(K. K. Desai & Talukdar, 2003)	Study design: Not experimental study design
123	Database search	(P. S. Desai, 2000)	Outcome: Interventions targeting sales of product that could not be categorized as healthy or unhealthy
124	Glanz, Bader & Iyer. (2012)	(Desmet & Renaudin, 1998)	Study design: Not experimental study design
125	Glanz, Bader & Iyer. (2012) + Hartman et al (2018)	(S. K. Dhar & Hoch, 1996)	Outcome: Interventions targeting sales of product that could not be categorized as healthy or unhealthy
126	Glanz, Bader & Iyer. (2012)	(R. Dhar & Wertenbroch, 2000)	Outcome: Interventions targeting sales of product that could not be categorized as healthy or unhealthy
127	Glanz, Bader & Iyer. (2012)	(S. K. Dhar, Hoch, & Kumar, 2001)	Publications: Articles published in non-peer reviewed journals
128	Cadario and Chardon, 2020	(Diliberti, Bordi, Conklin, Roe, & Rolls, 2004)	Setting: Not in real grocery store.
129	Cadario and Chardon, 2020	(DiSantis et al., 2013)	Setting: Not in real grocery store.
130	Database search	(Doble, Ang Jia Ler, & Finkelstein, 2020)	Outcome: Outcome not real change in consumption or purchase behavior
131	Cadario and Chardon, 2020	(Donnelly, Zatz, Svirsky, & John, 2018)	Setting: Not in real grocery store.
132	Escaron, Meinen, Nitzke & Martines-Donete. (2013) + Cameron (2016)	(Dougherty, Wittsten, & Guarino, 1990)	Study design: Not experimental study design
133	Cadario and Chardon, 2020	(Downs, Wisdom, Wansink, & Loewenstein, 2013)	Setting: Not in real grocery store.
134	Glanz, Bader & Iyer. (2012)	(Drèze & Hoch, 1998)	Outcome: Outcome not real change in consumption or purchase behavior
135	Database search	(A. C. Drichoutis, Lazaridis, & Nayga, 2005)	Study design: Not experimental study design
136	Database search	(A. C. Drichoutis, Lazaridis, & Nayga Jr, 2007)	Study design: Not experimental study design
137	Hersey et al. (2013)	(Andreas C Drichoutis, Lazaridis, & Nayga, 2009)	Setting: Not in real grocery store. Stimulated grocery store
138	Cadario and Chardon, 2020	(Dubbert, Johnson, Schlundt, & Montague, 1984)	Setting: Not in real grocery store.
139	Hartman et al (2018)	(Ducrot et al., 2016)	Setting: Not in real grocery store. Stimulated grocery store
140	Database search	(Duffett & Foster, 2017)	Study design: Not experimental study design
141	Afskin et al. (2017)	(Duffey et al., 2010)	Study design: Not experimental study design
142	Database search	(Dulsrud & Jacobsen, 2009)	Study design: Not experimental study design
143	Cadario and Chardon, 2020	(Dumanovsky et al., 2011)	Setting: Not in real grocery store.
144	Database search	(Duncan Herrington, 1996)	Study design: Not experimental study design
145	Database search	(Dunn, Dean, Johnson, Leidner, & Sharkey, 2012)	Study design: Not experimental study design
146	Cameron (2016)	(Dwivedi, Harvey, & Close, 1997)	Study design: Study did not fit study design criteria

147	Glanz, Bader & Iyer. (2012)	(Ebster, Wagner, & Neumueller, 2009)	Outcome: Outcome not real change in consumption or purchase behavior
148	Hersey et al. (2013)	(Edge MS., 2010)	Publications: Articles published in non-peer reviewed journals
149	Cadario and Chardon, 2020	(B. Elbel, Kersh, Brescoll, & Dixon, 2009)	Setting: Not in real grocery store.
150	Cadario and Chardon, 2020	(Brian Elbel, Gyamfi, & Kersh, 2011)	Setting: Not in real grocery store.
151	Cadario and Chardon, 2020	(Brian Elbel et al., 2013)	Setting: Not in real grocery store.
152	Database search	(Elberg, Gardete, Macera, & Noton, 2019)	Outcome: Interventions targeting sales of product that could not be categorized as healthy or unhealthy
153	Glanz, Bader & Iyer. (2012)	(C. Elliott, 2008)	Study design: Not experimental study design
154	Glanz, Bader & Iyer. (2012)	(C. D. Elliott, 2009)	Study design: Not experimental study design
155	Cadario and Chardon, 2020	(Ellison, Lusk, & Davis, 2013)	Setting: Not in real grocery store.
156	Hartman et al. (2018)	(Elofsson, Bengtsson, Matsdotter, & Arntyr, 2016)	Outcome: Interventions targeting sales of product that could not be categorized as healthy or unhealthy
157	Cadario and Chardon, 2020	(Elsbernd et al., 2016)	Setting: Not in real grocery store.
158	Cadario and Chardon, 2020	(Ensaff et al., 2015)	Setting: Not in real grocery store.
159	Epstein et al. (2012)	(Leonard H Epstein, Dearing, Handley, Roemmich, & Paluch, 2006)	Setting: Not in real grocery store. Stimulated grocery store
160	Hartman et al. (2018) + Database search	(Leonard H Epstein et al., 2015)	Setting: Not in real grocery store. Stimulated grocery store
161	Cameron (2016)	(L. H. Epstein et al., 2016)	Setting: Not in real grocery store. Stimulated grocery store
162	Epstein et al. (2012)	(Leonard H Epstein, Handley, et al., 2006)	Setting: Not in real grocery store. Stimulated grocery store
163	Epstein et al. (2012)	(Leonard H Epstein, Dearing, Paluch, Roemmich, & Cho, 2007)	Setting: Not in real grocery store. Stimulated grocery store
164	Epstein et al. (2012)	(Leonard H Epstein, Dearing, Roba, & Finkelstein, 2010)	Setting: Not in real grocery store. Stimulated grocery store
165	Database search	(Escaron, Meinen, Nitzke, & Martinez-Donate, 2013)	Study design: Review article
166	Hersey et al. (2013)	(Feunekes, Gortemaker, Willems, Lion, & Van Den Kommer, 2008)	Outcome: Outcome not real change in consumption or purchase behavior
167	Cadario and Chardon, 2020	(Finkelstein, Strombotne, Chan, & Krieger, 2011)	Setting: Not in real grocery store.
168	Database search	(Finnell, John, & Thompson, 2017)	Setting: Not in real grocery store.
169	Afskin et al. (2017)	(Fletcher, Frisvold, & Tefft, 2010)	Intervention: Not in-store intervention meeting criteria
170	Escaron, Meinen, Nitzke & Martines-Donete. (2013)	(Foerster et al., 1998)	Intervention: Not in-store intervention meeting criteria
171	Escaron, Meinen, Nitzke & Martines-Donete. (2013)	(Foerster et al., 1995)	Wrong study setting: Study did not evaluate a grocery store intervention but a public "5 a day campaign" impact on fruits and vegetable consumption

172	Cameron (2016)	(Forwood, Ahern, Marteau, & Jebb, 2015)	Setting: Not in real grocery store. Stimulated grocery store
173	Glanz, Bader & Iyer. (2012)	(Franco et al., 2009)	Outcome: Interventions targeting sales of product that could not be categorized as healthy or unhealthy
174	Adam (2016) + Hersey et al. (2013)	(M. R. Freedman & Connors, 2010)	Study design: Not experimental study design
175	Cadario and Chardon, 2020	(M. R. Freedman & Larner, 2011)	Setting: Not in real grocery store.
176	Cadario and Chardon, 2020	(M. R. Freedman & Brochado, 2010)	Setting: Not in real grocery store.
177	Adam (2016)	(D. A. Freedman, Bell, & Collins, 2011)	Setting: Not in real grocery store.
178	Database search	(Freire & Rudkin, 2019)	Study design: Not experimental study design
179	Epstein et al. (2012) + Afskin et al. (2017)	(Simone A French, Jeffery, Story, Hannan, & Snyder, 1997)	Setting: Not in real grocery store.
180	Epstein et al. (2012) + Afskin et al. (2017)	(Simone A French, Story, et al., 1997)	Setting: Not in real grocery store.
181	Afskin et al. (2017)	(Simone A French et al., 2010)	Setting: Not in real grocery store.
182	Epstein et al. (2012) + Liberato, Bailie & Brimblecombe. (2014) + Afshin et al. (2017)	(S. A. French et al., 2001)	Setting: Not in real grocery store.
183	Cadario and Chardon, 2020	(Friis et al., 2017)	Setting: Not in real grocery store.
184	Cadario & Chardon, 2020	(Gaigi et al., 2015)	Publications: Non-English Language publications
185	Cadario and Chardon, 2020	(Geaney et al., 2016)	Setting: Not in real grocery store.
186	Epstein et al, (2012)	(J. C. Giesen, Havermans, Nederkoorn, & Jansen, 2012)	Setting: Not in real grocery store. Stimulated grocery store
187	Epstein et al. (2012)	(Janneke CAH Giesen, Payne, Havermans, & Jansen, 2011)	Setting: Not in real grocery store. Stimulated grocery store
188	Database search	(Gill & Rudkin, 2014)	Setting: Not in real grocery store.
189	Database search + Liberato, Bailie & Brimblecombe. (2014) + Adam (2016)	(Joel Gittelsohn et al., 2010)	Outcome: Outcome not real change in consumption or purchase behavior
190	Adam (2016) + Escaron, Meinen, Nitzke & Martines-Donete. (2013)	(Gittelsohn, Kim, He, & Pardilla, 2013)	Outcome: Outcome not real change in consumption or purchase behavior
191	Adam (2016) + Glanz, Bader & Iyer. (2012) + Escaron, Meinen, Nitzke & Martines-Donete. (2013)	(J. Gittelsohn et al., 2006)	Study design: Not experimental study design
192	Adam (2016) + Glanz, Bader & Iyer. (2012) + Liberato, Bailie & Brimblecombe. (2014)	(J. Gittelsohn, V. Vijayadeva, et al., 2010)	Outcome: Outcome not real change in consumption or purchase behavior
193	Escaron, Meinen, Nitzke & Martines-Donete (2013)	(Joel Gittelsohn et al., 2006)	Study design: Not experimental study design
194	Adam (2016) + Escaron, Meinen, Nitzke & Martines-Donete. (2013)	(J. Gittelsohn, S.i Suratkar, et al., 2010)	Study design: Not experimental study design
195	Database search	(Glanz & Yaroch, 2004)	Study design: Review article
196	Database search	(Gopalan et al., 2019)	Setting: Not in real grocery store.
197	Afskin et al. (2017)	(Gordon-Larsen, Guilkey, & Popkin, 2011)	Setting: Not in real grocery store.

198	Hersey et al. (2013)	(Gorton, Mhurchu, Chen, & Dixon, 2009)	Intervention: Not in-store intervention meeting criteria
199	Cadario and Chardon, 2020	(Goto, Waite, Wolff, Chan, & Giovanni, 2013)	Setting: Not in real grocery store.
200	Hersey et al. (2013)	(Green, 2006)	Publications: Articles published in non-peer reviewed journals
201	Cadario and Chardon, 2020	(Greene, Gabrielyan, Just, & Wansink, 2017)	Setting: Not in real grocery store.
202	Hersey et al. (2013)	(Grunert, Wills, & Fernández-Celemín, 2010b)	Outcome: Interventions targeting sales of product that could not be categorized as healthy or unhealthy
203	Hersey et al. (2013)	(Grunert, Fernández-Celemín, Wills, genannt Bonsmann, & Nureeva, 2010a)	Study design: Not experimental study design
204	Database search	(Guan, Atlas, & Vadiveloo, 2018)	Setting: Not in real grocery store.
205	Database search	(Hamlin & McNeill, 2016)	Outcome: Outcome not real change in consumption or purchase behavior
206	Cadario and Chardon 2020	(Andrew S Hanks, Just, Smith, & Wansink, 2012)	Setting: Not in real grocery store.
207	Cadario and Chardon, 2020	(A. S. Hanks, Just, & Wansink, 2013)	Setting: Not in real grocery store.
208	Hersey et al. (2013)	(Guiding Stars Licensing Company, 2010b)	Publications: Articles published in non-peer reviewed journals
209	Epstein et al. (2012)	(Harnack & French, 2008)	Setting: Not in real grocery store.
210	Glanz, Bader & Iyer (2012)	(J. L. Harris, Schwartz, & Kelly, 2009)	Publications: Articles published in non-peer reviewed journals
211	Glanz, Bader & Iyer (2012)	(Jennifer L Harris, Schwartz, & Brownell, 2010)	Study design: Not experimental study design
212	Database search	(Hartmann-Boyce et al., 2018)	Study design: Review article
213	Glanz, Bader & Iyer (2012)	(Hawkes, 2008)	Study design: Not experimental study design
214	Glanz, Bader & Iyer (2012)	(Hawkes, 2010)	Study design: Not experimental study design
215	Glanz, Bader & Iyer (2012)	(Heilman, Nakamoto, & Rao, 2002)	Outcome: Interventions targeting sales of product that could not be categorized as healthy or unhealthy
216	Database search + Afskin et al. (2017) + Adam (2016) + Liberato, Bailie & Brimblecombe (2014)	(D. R. Herman, Harrison, Afifi, & Jenks, 2008)	Setting: Not in real grocery store.
217	Adam (2016) + Epstein et al. (2012)	(Dena R Herman, Harrison, & Jenks, 2006)	Outcome: Outcome not real change in consumption or purchase behavior
218	Adam (2016) + Glanz, Bader & Iyer. (2012) + Escaron, Meinen, Nitzke & Martines-Donete. (2013)	(Ho et al., 2008)	Study design: Study did not fit study design criteria
219	Glanz, Bader & Iyer. (2012)	(Hoch, Bradlow, & Wansink, 1999)	Study design: Not experimental study design
220	Cadario and Chardon, 2020	(Hoefkens, Lachat, Kolsteren, Van Camp, & Verbeke, 2011)	Setting: Not in real grocery store.
221	Glanz, Bader & Iyer (2012)	(Holweg, Schnedlitz, & Teller, 2009)	Outcome: Outcome not real change in consumption or purchase behavior

222	Epstein et al. (2012) + Afskin et al. (2017)	(Horgen & Brownell, 2002)	Setting: Not in real grocery store.
223	Cadario and Chardon 2020	(Hubbard et al., 2015)	Setting: Not in real grocery store.
224	Glanz, Bader & Iyer (2012)	(Huddleston, Whipple, Nye Mattick, & Jung Lee, 2009)	Setting: Not in real grocery store.
225	Glanz, Bader & Iyer (2012)	(Hui, Bradlow, & Fader, 2009)	Study design: Not experimental study design
226	Database search	(Huitink, Poelman, van den Eynde, Seidell, & Dijkstra, 2020)	Study design: Study did not fit study design criteria
227	Glanz, Bader & Iyer. (2012)	(Hunter, 2002)	Study design: Not experimental study design
228	Escaron, Meinen, Nitzke & Martines-Donete. (2013) + Hersey et al. (2013)	(Hunt et al., 1990)	Study design: Not experimental study design
229	Glanz, Bader & Iyer. (2012)	(Inman, Winer, & Ferraro, 2009)	Study design: Not experimental study design
230	Glanz, Bader & Iyer. (2012)	(Iyengar & Lepper, 2000)	Setting: Not in real grocery store.
231	Epstein et al. (2012) + Afskin et al. (2017)	(Jeffery, French, Raether, & Baxter, 1994)	Setting: Not in real grocery store.
232	Glanz, Bader & Iyer. (2012)	(Jensen, 1995)	Outcome: Outcome not real change in consumption or purchase behavior
233	Hersey et al. (2013)	(Jones & Richardson, 2007)	Outcome: Interventions targeting sales of product that could not be categorized as healthy or unhealthy
234	Afshin et al. 2017	(Jue et al., 2012)	Setting: Not in real grocery store.
235	Database search	(Julia et al., 2016)	Setting: Not in real grocery store.
236	Glanz, Bader & Iyer. (2012)	(Kahn & Wansink, 2004)	Setting: Not in real grocery store.
237	Hersey et al. (2013)	(Kelly et al., 2009)	Outcome: Outcome not real change in consumption or purchase behavior
238	Adam (2016)	(Kennedy et al., 2009)	Population: Population enrolled in study at health centers or other settings not directedly related to grocery store setting.
239	Afskin et al. (2017)	(Khan, Powell, & Wada, 2012)	Setting: Not in real grocery store.
240	Afskin et al. (2017)	(Kocken et al., 2012)	Setting: Not in real grocery store.
241	Afskin et al. (2017)	(Kottke, Pronk, Katz, Tillema, & Flottemesch, 2013)	Setting: Not in real grocery store.
242	Database search	(Koutoukidis et al., 2019)	Setting: Not in real grocery store.
243	Glanz, Bader & Iyer. (2012)	(Kozup, Creyer, & Burton, 2003)	Outcome: Outcome not real change in consumption or purchase behavior
244	Database search	(Kral, Bannon, & Moore, 2016)	Setting: Not in real grocery store.
245	Cadario and Chardon, 2020	(Krieger et al., 2013)	Setting: Not in real grocery store.
246	Glanz, Bader & Iyer. (2012)	(Krishna & Zhang, 1999)	Outcome: Interventions targeting sales of product that could not be categorized as healthy or unhealthy
247	Glanz, Bader & Iyer. (2012)	(Krishnan & Rao, 1995)	Outcome: Interventions targeting sales of product that could not be categorized as healthy or unhealthy
248	Cadario and Chardon, 2020	(Kroese, Marchiori, & de Ridder, 2016)	Setting: Not in real grocery store.
249	Cadario and Chardon, 2020	(Lachat et al., 2009)	Setting: Not in real grocery store.

250	Escaron, Meinen, Nitzke & Martines-Donete. (2013) + Hersey et al. (2013)	(Lang, Mercer, Tran, & Mosca, 2000)	Outcome: Interventions targeting sales of product that could not be categorized as healthy or unhealthy
251	Glanz, Bader & Iyer. (2012)	(N. Larson & Story, 2009)	Study design: Not experimental study design
252	Glanz, Bader & Iyer. (2012)	(R. Larson, 2005)	Study design: Not experimental study design
253	Glanz, Bader & Iyer. (2012)	(R. Larson, 2006)	Study design: Not experimental study design
254	Glanz, Bader & Iyer. (2012)	(N. I. Larson, Story, & Nelson, 2009)	Study design: Review article
255	Hersey et al. (2013)	(Larsson, Lissner, & Wilhelmsen, 1999)	Outcome: Interventions targeting sales of product that could not be categorized as healthy or unhealthy
256	Database search	(Le et al., 2016)	Duplicates: Duplicate publications with Ball et al. 2015 included in review
257	Glanz, Bader & Iyer. (2012)	(Lemon & Nowlis, 2002)	Study design: Not experimental study design
258	Hartman et al. (2018)	(Lent et al., 2014)	Setting: Not in real grocery store.
259	Cadario and Chardon, 2020	(Levin, 1996)	Setting: Not in real grocery store.
260	Escaron, Meinen, Nitzke & Martines-Donete. (2013)	(Alan S Levy, Schucker, Tenney, & Mathews, 1988)	Outcome: Outcome not real change in consumption or purchase behavior
261	Escaron, Meinen, Nitzke & Martines-Donete. (2013) + Jonathan van´t Riet. (2012) + Liberato, Bailie & Brimblecombe. (2014) + Cameron (2016)	(A. S. Levy, Mathews, Stephenson, Tenney, & Schucker, 1985)	Study design: Not experimental study design
262	Cadario and Chardon, 2020	(D. E. Levy, Riis, Sonnenberg, Barraclough, & Thorndike, 2012)	Setting: Not in real grocery store.
263	Glanz, Bader & Iyer. (2012)	(Lichtenstein, Netemeyer, & Burton, 1995)	Study design: Not experimental study design
264	Escaron, Meinen, Nitzke & Martines-Donete. (2013)	(Light et al., 1989)	Study design: Not experimental study design
265	Hersey et al. (2013)	(Lin C-TJ & Levy A., 2010)	Publications: Articles published in non-peer reviewed journals
266	Epstein et al. (2012) + Afskin et al. (2017)	(Lowe et al., 2010)	Setting: Not in real grocery store.
267	Hartman et al. (2018)	(Ma et al., 2016)	Intervention: Not in-store intervention meeting criteria
268	Hersey et al. (2013)	(Malam et al., 2009)	Publications: Articles published in non-peer reviewed journals
269	Adam (2016) + Cameron (2016)	(Martínez-Donate et al., 2015)	Intervention: Not in-store intervention meeting criteria
270	Cadario and Chardon, 2020	(Mayer et al., 1986)	Setting: Not in real grocery store.
271	Hersey et al. (2013)	(Maubach, Hoek, & McCreanor, 2009b)	Publications: Articles published in non-peer reviewed journals
272	Cadario and Chardon, 2020	(Mazza, Dynan, Siegel, & Tucker, 2018)	Setting: Not in real grocery store.
273	Afskin et al. (2017)	(Meyer et al., 2014)	Study design: Not experimental study design
274	Cadario and Chardon, 2020	(Meyers & Stunkard, 1980)	Setting: Not in real grocery store.
275	Adam (2016) + Escaron, Meinen, Nitzke & Martines-Donete. (2013)	(Cliona Ni Mhurchu et al., 2007)	Study design: Not experimental study design
276	Escaron, Meinen, Nitzke & Martines-Donete. (2013)	(C Ni Mhurchu et al., 2009)	Study design: Not experimental study design

277	Epstein et al. (2012) + Afskin et al. (2017)	(Michels, Bloom, Riccardi, Rosner, & Willett, 2008)	Setting: Not in real grocery store.
278	Glanz, Bader & Iyer. (2012)	(E. G. Miller & Kahn, 2005)	Outcome: Outcome not real change in consumption or purchase behavior
279	Cadario and Chardon, 2020	(G. F. Miller, Gupta, Kropp, Grogan, & Mathews, 2016)	Setting: Not in real grocery store.
280	Cadario and Chardon, 2020	(Mishra, Mishra, & Masters, 2012)	Setting: Not in real grocery store.
281	Cadario and Chardon, 2020	(Mollen, Rimal, Ruiter, & Kok, 2013)	Setting: Not in real grocery store.
282	Glanz, Bader & Iyer. (2012)	(Morales, Kahn, McAlister, & Broniarczyk, 2005)	Setting: Not in real grocery store. Stimulated grocery store
283	Glanz, Bader & Iyer. (2012)	(Moreau, Krishna, & Harlam, 2002)	Outcome: Interventions targeting sales of product that could not be categorized as healthy or unhealthy
284	Cadario and Chardon, 2020	(Morizet, Depezay, Combris, Picard, & Giboreau, 2012)	Setting: Not in real grocery store.
285	Glanz, Bader & Iyer. (2012)	(Morton & Zettelmeyer, 2004)	Study design: Not experimental study design
286	Jonathan van´t Riet. (2012) + Cameron (2016)	(Thomas E Muller, 1984)	Outcome: Outcome not real change in consumption or purchase behavior
287	Cameron (2016)	(T. E. Muller, 1984)	Study design: Study did not fit study design criteria
288	Escaron, Meinen, Nitzke & Martines-Donete. (2013) + Jonathan van´t Riet. (2012) + Åpent Søk	(R. Mullis & Pirie, 1988)	Outcome: Outcome not real change in consumption or purchase behavior
289	Escaron, Meinen, Nitzke & Martines-Donete. (2013) + Cameron (2016)	(R. M. Mullis et al., 1987)	Outcome: Outcome not real change in consumption or purchase behavior
290	Cameron (2016)	(Nakamura, Pechey, Suhrcke, Jebb, & Marteau, 2014)	Study design: Not experimental study design
291	Hersey et al. (2013)	(The National Heart Foundation of Australia, 2009)	Publications: Articles published in non-peer reviewed journals
292	Epstein et al. (2012) + Hartman et al. (2018)	(Nederkoorn, Havermans, Giesen, & Jansen, 2011)	Setting: Not in real grocery store. Stimulated grocery store
293	Database search	(Nguyen, Emberger-Klein, & Menrad, 2019)	Outcome: Interventions targeting sales of product that could not be categorized as healthy or unhealthy
294	Hartman et al. (2018)	(Ni Mhurchu et al., 2017)	Intervention: Not in-store intervention meeting criteria
295	Database search + Cameron (2016)	(Nikolova & Inman, 2015)	Study design: Not experimental study design
296	Adam (2016)	(Novotny et al., 2011)	Study design: Not experimental study design
297	Database search	(Närhinen, Nissinen, & Puska, 2000)	Study design: Study did not fit study design criteria
298	Escaron, Meinen, Nitzke & Martines-Donete. (2013)	(O'Loughlin, Ledoux, Barnett, & Paradis, 1996)	Outcome: Interventions targeting sales of product that could not be categorized as healthy or unhealthy

299	Escaron, Meinen, Nitzke & Martines-Donete. (2013)	(O'Loughlin, Renaud, Richard, Gomez, & Paradis, 1998)	Outcome: Interventions targeting sales of product that could not be categorized as healthy or unhealthy
300	Escaron, Meinen, Nitzke & Martines-Donete. (2013)	(Odenkirchen et al., 1992)	Study design: Not experimental study design
301	Adam (2016) + Escaron, Meinen, Nitzke & Martines-Donete. (2013) + Cadario & Chardon, 2020 + Cameron (2016)	(Ogawa et al., 2011)	Study design: Study did not fit study design criteria
302	Escaron, Meinen, Nitzke & Martines-Donete. (2013) + Jonathan van´t Riet. (2012) + Cameron (2016)	(Olson, Bisogni, & Thonney, 1982)	Outcome: Outcome not real change in consumption or purchase behavior
303	Cadario and Chardon, 2020	(Dana Lee Olstad, Goonewardene, McCargar, & Raine, 2014)	Setting: Not in real grocery store.
304	Cadario and Chardon, 2020	(D. L. Olstad, Vermeer, McCargar, Prowse, & Raine, 2015)	Setting: Not in real grocery store.
305	Database search + Afskin et al. (2017) + Escaron, Meinen, Nitzke & Martines-Donete. (2013) + Cameron (2016)	(Paine-Andrews, Francisco, Fawcett, Johnston, & Coen, 1997)	Outcome: Outcome not real change in consumption or purchase behavior
306	Escaron, Meinen, Nitzke & Martines-Donete. (2013)	(Paradis et al., 1995)	Setting: Not in real grocery store.
307	Database search	(Paton, Smith, Fraser, & McCormack, 1996)	Outcome: Outcome not real change in consumption or purchase behavior
308	Glanz, Bader & Iyer. (2012)	(Pauwels & Srinivasan, 2004)	Outcome: Interventions targeting sales of product that could not be categorized as healthy or unhealthy
309	Database search + Cameron (2016)	(Payne, Niculescu, Just, & Kelly, 2015)	Outcome: Outcome not real change in consumption or purchase behavior
310	Database search	(Payne Riches, Aveyard, Piernas, Rayner, & Jebb, 2019)	Setting: Not in real grocery store.
311	Cadario and Chardon, 2020	(Perry et al., 2004)	Setting: Not in real grocery store.
312	Database search + Liberato, Bailie & Brimblecombe. (2014) + Adam (2016)	(Phipps et al., 2015)	Setting: Not in real grocery store.
313	Database search	(Phipps et al., 2014)	Study design: Not experimental study design
314	Cadario and Chardon, 2020	(Policastro, Smith, & Chapman, 2017)	Setting: Not in real grocery store.
315	Cadario and Chardon, 2020	(Policastro, Palm, Schwartz, & Chapman, 2017)	Setting: Not in real grocery store.
316	Afskin et al. (2017)	(L. M. Powell & Han, 2011)	Setting: Not in real grocery store.
317	Afskin et al. (2017)	(Lisa M Powell & Bao, 2009)	Setting: Not in real grocery store.
318	Escaron, Meinen, Nitzke & Martines-Donete. (2013)	(Probart, 1993)	Outcome: Outcome not real change in consumption or purchase behavior
319	Glanz, Bader & Iyer. (2012)	(Healthy Corner Stores Network, 2009)	Publications: Articles published in non-peer reviewed journals

320	Cadario and Chardon, 2020	(Pulos & Leng, 2010)	Setting: Not in real grocery store.
321	Adam (2016) + Akshin et al. 2017	(Ruopeng An, Patel, Segal, & Sturm, 2013)	Setting: Not in real grocery store.
322	Glanz, Bader & Iyer. (2012)	(Raju, Sethuraman, & Dhar, 1995)	Study design: Not experimental study design
323	Cadario and Chardon, 2020	(Redden et al., 2015)	Setting: Not in real grocery store.
324	Escaron, Meinen, Nitzke & Martines-Donete. (2013) + Jonathan van´t Riet. (2012)	(Reger, Wootan, Booth-Butterfield, & Smith, 1998)	Setting: Not in real grocery store.
325	Escaron, Meinen, Nitzke & Martines-Donete. (2013)	(Nash, Booth-Butterfield, & Cooper, 2005)	Setting: Not in real grocery store.
326	Escaron, Meinen, Nitzke & Martines-Donete. (2013) + Liberato, Bailie & Brimblecombe. (2014)	(Reger, Wootan, & Booth-Butterfield, 2000)	Intervention: Not in-store intervention meeting criteria
327	Cadario and Chardon, 2020	(Reicks, Redden, Mann, Mykerezi, & Vickers, 2012)	Setting: Not in real grocery store.
328	Glanz, Bader & Iyer. (2012)	(Reicks, Splett, & Fishman, 1997)	Publications: Articles published in non-peer reviewed journals
329	Hersey et al. (2013)	(Reid, D'Angelo, Dombrow, Heshka, & Dean, 2004)	Study design: Not experimental study design
330	Glanz, Bader & Iyer. (2012)	(Rettie & Brewer, 2000)	Outcome: Outcome not real change in consumption or purchase behavior
331	Cadario and Chardon, 2020	(Roberto, Larsen, Agnew, Baik, & Brownell, 2010)	Setting: Not in real grocery store.
332	Escaron, Meinen, Nitzke & Martines-Donete. (2013) + Jonathan van´t Riet. (2012) + Liberato, Bailie & Brimblecombe. (2014) + Escaron, Meinen, Nitzke & Martines-Donete. (2013) + Jonathan van´t Riet. (2012) + Liberato, Bailie & Brimblecombe. (2014) + Cameron (2016)	(Rodgers et al., 1994)	Study design: Not experimental study design
333	Glanz, Bader & Iyer. (2012)	(Rose et al., 2009)	Study design: Not experimental study design
334	Adam (2016)	(Rosecrans et al., 2008)	Study design: Not experimental study design
335	Cadario and Chardon, 2020	(Rozin et al., 2011)	Setting: Not in real grocery store.
336	Hartman et al. (2018) + Cameron (2016)	(Russo, Staelin, Nolan, Russell, & Metcalf, 1986)	Study design: Not experimental study design
337	Cameron (2016)	(Sacks, Tikellis, Millar, & Swinburn, 2011)	Study design: Study did not fit study design criteria
338	Glanz, Bader & Iyer. (2012)	(Samuels et al., 2010)	Study design: Not experimental study design
339	Database search	(Sanchez-Flack et al., 2017)	Dobbel study. Ayala et al. 2013 included in review
340	Cameron (2016)	(Schucker, Levy, Tenney, & Mathews, 1992)	Study design: Not experimental study design
341	Database search	(Schultz & Litchfield, 2016)	Outcome: Outcome not real change in consumption or purchase behavior
342	Cadario and Chardon, 2020	(M. B. Schwartz, 2007)	Setting: Not in real grocery store.
343	Cadario and Chardon, 2020	(J. Schwartz, Riis, Elbel, & Ariely, 2012)	Setting: Not in real grocery store.

344	Hersey et al. (2013)	(V. Scott & Worsley, 1994)	Outcome: Outcome not real change in consumption or purchase behavior
345	Escaron, Meinen, Nitzke & Martines-Donete. (2013) + Cameron (2016)	(J. A. Scott, Begley, Miller, & Binns, 1991)	Outcome: Outcome not real change in consumption or purchase behavior
346	Cadario and Chardon, 2020	(Shah, Bettman, Ubel, Keller, & Edell, 2014)	Setting: Not in real grocery store.
347	Escaron, Meinen, Nitzke & Martines-Donete. (2013)	(Shannon, Mullis, Pirie, & Pheley, 1990)	Study design: Not experimental study design
348	Escaron, Meinen, Nitzke & Martines-Donete. (2013)	(Sharma et al., 2009)	Study design: Not experimental study design
349	Adam (2016)	(Sigurdsson, Larsen, & Gunnarsson, 2014)	Study design: Not experimental study design
350	Glanz, Bader & Iyer. (2012)	(Silayoi & Speece, 2007)	Outcome: Outcome not real change in consumption or purchase behavior
351	Glanz, Bader & Iyer. (2012)	(Sirohi, McLaughlin, & Wittink, 1998)	Outcome: Outcome not real change in consumption or purchase behavior
352	Hartman et al. (2018)	(Smith, Parnell, Brown, & Gray, 2013)	Outcome: Interventions targeting sales of product that could not be categorized as healthy or unhealthy
353	Database search	(Smith-Drelich, 2016)	Outcome: Outcome not real change in consumption or purchase behavior
354	Adam (2016) + Escaron, Meinen, Nitzke & Martines-Donete (2013) + Jonathan van´t Riet. (2012) + Liberato, Bailie & Brimblecombe (2014)	(H.-J. Song et al., 2009)	Setting: Not in real grocery store.
355	Adam (2016)	(Hee-Jung Song et al., 2011)	Study design: Not experimental study design
356	Escaron, Meinen, Nitzke & Martines-Donete. (2013)	(Soriano & Dozier, 1978)	Study design: Not experimental study design
357	Adam (2016)	(I Steenhuis, Van Assema, Reubsaet, & Kok, 2004)	Study design: Not experimental study design
358	Adam (2016) + Escaron, Meinen, Nitzke & Martines-Donete. (2013) + Hersey et al. (2013)	(Ingrid Steenhuis, van Assema, van Breukelen, & Glanz, 2004)	Outcome: Outcome not real change in consumption or purchase behavior
359	Hersey et al. (2013)	(IHM Steenhuis et al., 2010)	Setting: Not in real grocery store.
360	Cadario and Chardon, 2020	(I. Steenhuis et al., 2004)	Setting: Not in real grocery store.
361	Cameron (2016)	(Surkan, Tabrizi, Lee, Palmer, & Frick, 2016)	Study design: Study did not fit study design criteria
362	Adam (2016) + Hersey et al. (2013)	(Sutherland, Kaley, & Fischer, 2010)	Study design: Not experimental study design
363	Hersey et al. (2013)	(Synovate, 2005)	Publications: Articles published in non-peer reviewed journals
364	Database search	(Saarela, 2014)	Intervention: Not in-store intervention meeting criteria
365	Cadario & Chardon, 2020	(Tal & Wansink, 2015)	Study design: Study did not fit study design criteria
366	Cadario and Chardon, 2020	(Tandon et al., 2011)	Setting: Not in real grocery store.
367	Jonathan van´t Riet. (2012) + Cameron (2016)	(Teisl & Levy, 1997)	Study design: Study did not fit study design criteria
368	Cameron (2016)	(Thapa et al., 2014)	Publications: Articles published in non-peer reviewed journals
369	Cadario and Chardon, 2020	(Thomas et al., 2017)	Setting: Not in real grocery store.

370	Database search	(A. Thorndike, Levy, Macias-Navarro, Franckle, & Rimm, 2015)	Setting: Not in real grocery store.
371	Cadario and Chardon, 2020	(A. N. Thorndike, Sonnenberg, Riis, Barraclough, & Levy, 2012)	Setting: Not in real grocery store.
372	Cadario and Chardon, 2020	(A. N. Thorndike, Riis, Sonnenberg, & Levy, 2014)	Setting: Not in real grocery store.
373	Database search	(Thow, Downs, & Jan, 2014)	Study design: Review article
374	Cadario and Chardon, 2020	(Turnwald, Boles, & Crum, 2017)	Setting: Not in real grocery store.
375	Database search	(Vadiveloo et al., 2020)	Setting: Not in real grocery store.
376	Escaron, Meinen, Nitzke & Martines-Donete. (2013)	(van Assema, Brug, Glanz, Dolders, & Mudde, 1998)	Study design: Not experimental study design
377	Escaron, Meinen, Nitzke & Martines-Donete. (2013)	(Van Assema et al., 2006)	Setting: Not in real grocery store.
378	Cadario and Chardon, 2020	(Van Ittersum & Wansink, 2013)	Setting: Not in real grocery store.
379	Glanz, Bader & Iyer. (2012)	(Ellen Van Kleef, Van Trijp, Paeps, & Fernández-Celemín, 2008)	Outcome: Outcome not real change in consumption or purchase behavior
380	Cadario and Chardon, 2020	(E. van Kleef, van den Broek, & van Trijp, 2015)	Setting: Not in real grocery store.
381	Database search	(Vandenbroele, Slabbinck, Van Kerckhove, & Vermeir, 2018)	Study design: Study did not fit study design criteria
382	Cadario and Chardon, 2020	(Vanderlee & Hammond, 2014)	Setting: Not in real grocery store.
383	Cadario and Chardon, 2020	(Vasiljevic et al., 2018)	Setting: Not in real grocery store.
384	Database search	(Volkova et al., 2014)	Study design: Not experimental study design
385	Database search	(von Philipsborn et al., 2019)	Study design: Review article
386	Hersey et al. (2013)	(Vyth et al., 2010)	Outcome: Outcome not real change in consumption or purchase behavior
387	Glanz, Bader & Iyer. (2012)	(Vyth et al., 2009)	Outcome: Outcome not real change in consumption or purchase behavior
388	Escaron, Meinen, Nitzke & Martines-Donete. (2013) + Cameron (2016)	(Wagner, Winett, & Walbert-Rankin, 1992)	participants part of a Nutrition for a Lifesime System program
389	Glanz, Bader & Iyer. (2012)	(Walters & Jamil, 2002)	Intervention: Not in-store intervention meeting criteria
390	Glanz, Bader & Iyer. (2012)	(Wanke, Lehmann, & Bless, 1997)	Study design: Not experimental study design
391	Cadario and Chardon, 2020	(Wansink & Chandon, 2014)	Setting: Not in real grocery store.
392	Cadario and Chardon, 2020	(Wansink & Payne, 2007)	Setting: Not in real grocery store.
393	Cadario and Chardon, 2020	(Wansink & Van Ittersum, 2013)	Setting: Not in real grocery store.
394	Cadario and Chardon, 2020	(Wansink & Kim, 2005)	Setting: Not in real grocery store.
395	Cadario and Chardon, 2020	(Wansink, Van Ittersum, & Painter, 2006)	Setting: Not in real grocery store.
396	Cadario and Chardon, 2020	(Wansink & Hanks, 2013)	Setting: Not in real grocery store.

397	Cadario and Chardon, 2020	(Wansink, Van Ittersum, & Payne, 2014)	wrong setting not in grocery store
398	Cadario and Chardon, 2020	(Wansink, Bhana, Qureshi, & Cadenhead, 2016)	wrong setting not in grocery store
399	Hartman et al. (2018)	(Wansink, Soman, & Herbst, 2017)	Outcome: Outcome not real change in consumption or purchase behavior
400	Glanz, Bader & Iyer. (2012)	(Wansink, 1996)	Setting: Not in real grocery store.
401	Glanz, Bader & Iyer. (2012)	(Wansink, 2004)	Study design: Not experimental study design
402	Glanz, Bader & Iyer. (2012) + Cadario and Chardon, 2020	(Wansink & Chandon, 2006)	Setting: Not in real grocery store.
403	Glanz, Bader & Iyer. (2012)	(Wansink, Kent, & Hoch, 1998)	Intervention: Not in-store intervention meeting criteria
404	Glanz, Bader & Iyer. (2012)	(Wilma E Waterlander, Steenhuis, de Vet, Schuit, & Seidell, 2010)	Study design: Not experimental study design
405	Database search + Hartman et al. (2018)	(Wilma Elzeline Waterlander, Mhurchu, & Steenhuis, 2014)	Wrong setting: Visual supermarket
406	Hartman et al. (2018) + Database search	(Wilma E Waterlander, Steenhuis, de Boer, Schuit, & Seidell, 2013)	Setting: Not in real grocery store. Stimulated grocery store
407	Hartman et al. (2018)	(W. E. Waterlander, I. H. Steenhuis, M. R. de Boer, A. J. Schuit, & J. C. Seidell, 2012)	Study design: Not experimental study design
408	Hartman et al. (2018)	(W. E. Waterlander, I. H. M. Steenhuis, M. R. de Boer, A. J. Schuit, & J. C. Seidell, 2012)	Wrong setting: Visual supermarket
409	Cadario and Chardon, 2020	(Webb, Solomon, Sanders, Akiyama, & Crawford, 2011)	wrong setting not in grocery store
410	Afskin et al. (2017)	(Wendt & Todd, 2011)	Publications: Articles published in non-peer reviewed journals
411	Hersey et al. (2013)	(Which? Consumers' Association, 2006)	Publications: Articles published in non-peer reviewed journals
412	Cadario and Chardon, 2020	(Wilson, Just, Swigert, & Wansink, 2017)	Wrong setting: Visual supermarket
413	Liberato, Bailie & Brimblecombe. (2014) + Jonathan van´t Riet. (2012) + Hartman et al. (2018) + Cameron (2016)	(Winett et al., 1991)	Setting: Not in real grocery store.
414	Hartman et al. (2018)	(Winett, Kramer, Walker, Malone, & Lane, 1988)	Setting: Not in real grocery store.
415	Database search	(Winkler et al., 2016)	Study design: Not experimental study design
416	Epstein et al. (2012)	(Yang & Chiou, 2010)	Setting: Not in real grocery store.
417	Database search	(Yao, Oppewal, & Wang, 2020)	Outcome: Outcome not real change in consumption or purchase behavior
418	Glanz, Bader & Iyer. (2012)	(Zhang, Krishna, & Dhar, 2000)	Outcome: Outcome not real change in consumption or purchase behavior
419	Glanz, Bader & Iyer. (2012)	(Zielke & Toporowski, 2009)	Publications: Articles published in non-peer reviewed journals

Excluded articles

Adam, A., & Jensen, J. D. (2016). What is the effectiveness of obesity related interventions at retail grocery stores and supermarkets?—a systematic review. *BMC public health, 16*(1), 1247.

Adams, M. A., Pelletier, R. L., Zive, M. M., & Sallis, J. F. (2005). Salad bars and fruit and vegetable consumption in elementary schools: a plate waste study. *Journal of the American Dietetic Association, 105*(11), 1789-1792.

Afshin, A., Penalvo, J., Del Gobbo, L., Kashaf, M., Micha, R., Morrish, K., . . . Mozaffarian, D. (2015). CVD Prevention Through Policy: a Review of Mass Media, Food/Menu Labeling, Taxation/Subsidies, Built Environment, School Procurement, Worksite Wellness, and Marketing Standards to Improve Diet. *Current Cardiology Reports, 17*(11). doi:10.1007/s11886-015-0658-9

Ahmed, A. (2008). Marketing of halal meat in the United Kingdom: Supermarkets versus local shops. *British Food Journal, 110*(7), 655-670. doi:10.1108/00070700810887149

Ailawadi, K. L. (2001). The retail power-performance conundrum: What have we learned? *Journal of Retailing, 77*(3), 299-318.

Akgungor, S., Groppel-Klein, A., Koenigstorfer, J., Gulcan, Y., & Kustepeli, Y. (2016). The Impact of Nutrition Labels on Food Sales: An In-Store Experiment in a Turkish Supermarket. *Economia Agro-alimentare, 18*(2), 207-228. doi:http://www.francoangeli.it/riviste/sommario.asp?IDRivista=87&lingua=en

Alamsyah, D. P., Othman, N. A., & Mohammed, H. A. A. (2020). The awareness of environmentally friendly products: The impact of green advertising and green brand image. *Management Science Letters, 10*(9), 1961-1968. doi:10.5267/j.msl.2020.2.017

Almenar, E., Catala, R., Hernandez-Munoz, P., & Gavara, R. (2009). Optimization of an active package for wild strawberries based on the release of 2-nonanone. *Lwt-Food Science and Technology, 42*(2), 587-593. doi:10.1016/j.lwt.2008.09.009

An, R. (2013). Effectiveness of subsidies in promoting healthy food purchases and consumption: a review of field experiments. *Public health nutrition, 16*(7), 1215-1228. doi:https://dx.doi.org/10.1017/S1368980012004715

An, R., Patel, D., Segal, D., & Sturm, R. (2013). Eating better for less: a national discount program for healthy food purchases in South Africa. *American journal of health behavior, 37*(1), 56-61.

An, R., & Sturm, R. (2017). A cash-back rebate program for healthy food purchases in South Africa: Selection and program effects in self-reported diet patterns. *American Journal of Health Behavior, 41*(2), 152-162. doi:10.5993/AJHB.41.2.6

Anderson, E. T., & Simester, D. I. (2001). Are sale signs less effective when more products have them? *Marketing Science, 20*(2), 121-142. doi:10.1287/mksc.20.2.121.10194

Anderson, E. T., & Simester, D. I. (2004). Long-run effects of promotion depth on new versus established customers: Three field studies. *Marketing Science, 23*(1), 4-20. doi:10.1287/mksc.1030.0040

Anderson, J. V., Bybee, D. I., Brown, R. M., McLean, D. F., Garcia, E. M., Breer, M. L., & Schillo, B. A. (2001). 5 a day fruit and vegetable intervention improves consumption in a low income population. *Journal of the American Dietetic Association, 101*(2), 195-202.

Andorfer, V. A., & Liebe, U. (2015). Do information, price, or morals influence ethical consumption? A natural field experiment and customer survey on the purchase of Fair Trade coffee. *Social Science Research, 52*, 330-350. doi:10.1016/j.ssresearch.2015.02.007

Andreis, D. R., & Milan, G. S. (2016). A study on the influence of the Sensory Aspects of consumer behavior of a supermarket. *Espacios, 37*(12). Retrieved from https://www.scopus.com/inward/record.uri?eid=2-s2.0-84967185931&partnerID=40&md5=cd050135a93f4ee5f9164b54bf48b5d5

Andrews, J. C., Burton, S., & Kees, J. (2011). Is simpler always better? Consumer evaluations of front-of-package nutrition symbols. *Journal of Public Policy & Marketing, 30*(2), 175-190.

Andreyeva, T., Long, M. W., & Brownell, K. D. (2010). The impact of food prices on consumption: a systematic review of research on the price elasticity of demand for food. *American journal of public health, 100*(2), 216-222.

Andreyeva, T., Luedicke, J., Middleton, A. E., Long, M. W., & Schwartz, M. B. (2012). Positive influence of the revised Special Supplemental Nutrition Program for Women, Infants, and Children food packages on access to healthy foods. *Journal of the Academy of Nutrition and Dietetics, 112*(6), 850-858.

Ang, F. J. L., Agrawal, S., & Finkelstein, E. A. (2019). Pilot randomized controlled trial testing the influence of front-of-pack sugar warning labels on food demand. *BMC public health, 19*(1), 164. doi:https://dx.doi.org/10.1186/s12889-019-6496-8

Anic, I.-D., & Vouk, R. (2005). Exploring the Relationships between Store Patronage Motives and Purchasing Outcomes for Major Shopping Trips in the Croatian Grocery Retailing. *Ekonomski Pregled, 56*(9), 634-657. doi:http://hrcak.srce.hr/ekonomski-pregled?lang=en

Ankamah-Yeboah, I., Nielsen, M., & Nielsen, R. (2016). Price premium of organic salmon in Danish retail sale. *Ecological Economics, 122*, 54-60. doi:10.1016/j.ecolecon.2015.11.028

Anliker, J. A., Winne, M., & Drake, L. T. (1992). An evaluation of the Connecticut farmers' market coupon program. *Journal of nutrition education, 24*(4), 185-191.

Anzman-Frasca, S., Braun, A. C., Ehrenberg, S., Epstein, L. H., Gampp, A., Leone, L. A., . . . Tauriello, S. (2018). Effects of a randomized intervention promoting healthy children's meals on children's ordering and dietary intake in a quick-service restaurant. *Physiology & behavior, 192*, 109-117.

Arce, L. F. (2019). Comparative analysis of the nutritional quality of products intended for public children against public adult from packaging. *Revista espanola de salud publica, 93*. Retrieved from <Go to ISI>://WOS:000489662200001

Arce-Urriza, M., Cebollada, J., & Tarira, M. F. (2017). The Effect of Price Promotions on Consumer Shopping Behavior across Online and Offline Channels: Differences between Frequent and Non-frequent Shoppers. *Information Systems and e-Business Management, 15*(1), 69-87. doi:https://link.springer.com/journal/volumesAndIssues/10257

Aschemann-Witzel, J., & Aagaard, E. M. N. (2014). Elaborating on the attitude-behaviour gap regarding organic products: young Danish consumers and in-store food choice. *International Journal of Consumer Studies, 38*(5), 550-558. doi:10.1111/ijcs.12115

Asenslo, L., & Montero, A. (2008). Analysis of fresh fish labelling in Spanish fish retail shops. *Food Control, 19*(8), 795-799. doi:10.1016/j.foodcont.2007.08.005

Astuty, E. (2019). Building green purchasing behavior. *Journal of Advanced Research in Dynamical and Control Systems, 11*(3Special Issue), 798-808. Retrieved from https://www.scopus.com/inward/record.uri?eid=2-s2.0-85069473628&partnerID=40&md5=056674b396ed41bae2cda1772defc54d

Auchincloss, A. H., Mallya, G. G., Leonberg, B. L., Ricchezza, A., Glanz, K., & Schwarz, D. F. (2013). Customer responses to mandatory menu labeling at full-service restaurants. *American journal of preventive medicine, 45*(6), 710-719.

Azeem, M. M., Baker, D., Villano, R. A., Mounter, S., & Griffith, G. (2019). Response to stockout in grocery stores: A small city case in a changing competitive environment. *Journal of Retailing and Consumer Services, 49*, 242-252. doi:10.1016/j.jretconser.2019.04.001

Bailey, A. R., Shaw, G., Alexander, A., & Nell, D. (2010). Consumer behaviour and the life course: shopper reactions to self-service grocery shops and supermarkets in England c.1947-75. *Environment and Planning a-Economy and Space, 42*(6), 1496-1512. doi:10.1068/a42247

Bains, A., Pakseresht, M., Roache, C., Beck, L., Sheehy, T., Gittelsohn, J., . . . Sharma, S. (2014). Healthy Foods North improves diet among Inuit and Inuvialuit women of childbearing age in Arctic Canada. *Journal of Human Nutrition and Dietetics, 27*, 175-185.

Bakini Driss, F. E., Ben Lallouna Hafsia, H., & Zghal, M. (2008). L'impact d'un eclairage additionnel dans un point de vente sur les reactions comportementales du consommateur. (Impact of an Additional Lighting on the Consumer Behavioral Reactions. With English summary.). *La Revue des Sciences de Gestion, 43*(229), 41-49. doi:http://www.cairn.info/revue-des-sciences-de-gestion.htm

Balcombe, K., Fraser, I., & Di Falco, S. (2010). Traffic lights and food choice: A choice experiment examining the relationship between nutritional food labels and price. *Food Policy, 35*(3), 211-220.

Ball, K., McNaughton, S. A., Le, H., Andrianopoulos, N., Inglis, V., McNeilly, B., . . . Crawford, D. (2013). ShopSmart 4 Health - protocol of a skills-based randomised controlled trial promoting fruit and vegetable consumption among socioeconomically disadvantaged women. *BMC public health, 13*, 466. doi:10.1186/1471-2458-13-466

Ball, K., McNaughton, S. A., Le, H. N., Abbott, G., Stephens, L. D., & Crawford, D. A. (2016). ShopSmart 4 Health: results of a randomized controlled trial of a behavioral intervention promoting fruit and vegetable consumption among socioeconomically disadvantaged women. *The American Journal of Clinical Nutrition, 104*(2), 436-445.

Ball, K., McNaughton, S. A., Mhurchu, C. N., Andrianopoulos, N., Inglis, V., McNeilly, B., . . . Crawford, D. (2011). Supermarket Healthy Eating for Life (SHELf): protocol of a randomised controlled trial promoting healthy food and beverage consumption through price reduction and skill-building strategies. *BMC public health, 11*, 715. doi:10.1186/1471-2458-11-715

Bangia, D., & Palmer-Keenan, D. M. (2014). Grocery store podcast about omega-3 fatty acids influences shopping behaviors: a pilot study. *Journal of nutrition education and behavior, 46*(6), 616-620.

Barat, S., & Paswan, A. K. (2005). Do higher face-value coupons cost more than they are worth in increased sales? *Journal of Product and Brand Management, 14*(6), 379-386. doi:10.1108/10610420510624530

Bartholomew, J. B., & Jowers, E. M. (2006). Increasing frequency of lower-fat entrees offered at school lunch: an environmental change strategy to increase healthful selections. *Journal of the american dietetic association, 106*(2), 248-252.

Baskin, E., Gorlin, M., Chance, Z., Novemsky, N., Dhar, R., Huskey, K., & Hatzis, M. (2016). Proximity of snacks to beverages increases food consumption in the workplace: A field study. *Appetite, 103*, 244-248.

Batte, M. T., Hooker, N. H., Haab, T. C., & Beaverson, J. (2007). Putting Their Money Where Their Mouths Are: Consumer Willingness to Pay for Multi-ingredient, Processed Organic Food Products. *Food Policy, 32*(2), 145-159. doi:http://www.sciencedirect.com/science/journal/03069192

Becchetti, L., Salustri, F., & Scaramozzino, P. (2019). Making Information on CSR Scores Salient: A Randomized Field Experiment. *Oxford Bulletin of Economics and Statistics, 81*(6), 1193-1213. doi:10.1111/obes.12301

Bell, D. R., Corsten, D., & Knox, G. (2011). From point of purchase to path to purchase: How preshopping factors drive unplanned buying. *Journal of marketing, 75*(1), 31-45.

Bercik, J., Horska, E., Wang, R. W. Y., & Chen, Y. C. (2016). The impact of parameters of store illumination on food shopper response. *Appetite, 106*, 101-109. doi:10.1016/j.appet.2016.04.010

Bernales-Korins, M., Ang, I. Y. H., Khan, S., & Geliebter, A. (2017). Psychosocial Influences on Fruit and Vegetable Intake Following a NYC Supermarket Discount. *Obesity*
, 25(8), 1321-1328. doi:10.1002/oby.21876

Berning, J. P. (2014). The Effect of Breakfast Cereal Coupons on the Nutritional Quality of Household Purchases. *International Food and Agribusiness Management Review, 17*, 41-60. doi:http://ifama.org/page-18348

Berning, J. P., Chouinard, H. H., Manning, K. C., McCluskey, J. J., & Sprott, D. E. (2010). Identifying Consumer Preferences for Nutrition Information on Grocery Store Shelf Labels. *Food Policy, 35*(5), 429-436. doi:http://www.sciencedirect.com/science/journal/03069192

Berning, J. P., Chouinard, H. H., & McCluskey, J. J. (2011). Do positive nutrition shelf labels affect consumer behavior? Findings from a field experiment with scanner data. *American Journal of Agricultural Economics, 93*(2), 364-369.

Bezawada, R., Balachander, S., Kannan, P., & Shankar, V. (2009). Cross-category effects of aisle and display placements: a spatial modeling approach and insights. *Journal of marketing, 73*(3), 99-117.

Bialkova, S., Grunert, K. G., & van Trijp, H. (2020). From desktop to supermarket shelf: Eye-tracking exploration on consumer attention and choice. *Food Quality and Preference, 81*. doi:10.1016/j.foodqual.2019.103839

Bihan, H., Méjean, C., Castetbon, K., Faure, H., Ducros, V., Sedeaud, A., . . . Hercberg, S. (2012). Impact of fruit and vegetable vouchers and dietary advice on fruit and vegetable intake in a low-income population. *European journal of clinical nutrition, 66*(3), 369-375.

Biswas, D., & Szocs, C. (2019). The Smell of Healthy Choices: Cross-Modal Sensory Compensation Effects of Ambient Scent on Food Purchases. *Journal of Marketing Research, 56*(1), 123-141. doi:10.1177/0022243718820585

Biswas, D., Szocs, C., Chacko, R., & Wansink, B. (2017). Shining Light on Atmospherics: How Ambient Light Influences Food Choices. *Journal of Marketing Research, 54*(1), 111-123. doi:http://www.marketingpower.com/jmr

Black, J. L., & Macinko, J. (2008). Neighborhoods and obesity. *Nutrition Reviews, 66*(1), 2-20.

Blakely, T., Mhurchu, C. N., Jiang, Y., Matoe, L., Funaki-Tahifote, M., Eyles, H. C., . . . Rodgers, A. (2011). Do effects of price discounts and nutrition education on food purchases vary by ethnicity, income and education? Results from a randomised, controlled trial. *Journal of Epidemiology & Community Health*, jech. 2010.118588.

Blanck, H. M., Thompson, O. M., Nebeling, L., & Yaroch, A. L. (2011). Improving Fruit and Vegetable Consumption: Use of Farm-to-Consumer Venues Among US Adults. *Preventing chronic disease, 8*(2). Retrieved from <Go to ISI>://WOS:000298967900019

Blattberg, R. C., Briesch, R., & Fox, E. J. (1995). How promotions work. *Marketing Science, 14*(3_supplement), G122-G132.

Block, J. P., Chandra, A., McManus, K. D., & Willett, W. C. (2010). Point-of-purchase price and education intervention to reduce consumption of sugary soft drinks. *American journal of public health, 100*(8), 1427-1433.

Blomquist, J., Bartolino, V., & Waldo, S. (2015). Price Premiums for Providing Eco-labelled Seafood: Evidence from MSC-certified Cod in Sweden. *Journal of Agricultural Economics, 66*(3), 690-704. doi:10.1111/1477-9552.12106

Bodor, J. N., Rice, J. C., Farley, T. A., Swalm, C. M., & Rose, D. (2010). The association between obesity and urban food environments. *Journal of Urban Health, 87*(5), 771-781.

Bodor, J. N., Ulmer, V. M., Futrell Dunaway, L., Farley, T. A., & Rose, D. (2010). The Rationale behind Small Food Store Interventions in Low-Income Urban Neighborhoods: Insights from New Orleans–. *The Journal of nutrition, 140*(6), 1185-1188.

Bollinger, B., Leslie, P., & Sorensen, A. (2011). Calorie posting in chain restaurants. *American Economic Journal: Economic Policy, 3*(1), 91-128.

Bolton, R. N., & Shankar, V. (2003). An empirically derived taxonomy of retailer pricing and promotion strategies. *Journal of Retailing, 79*(4), 213-224. doi:10.1016/j.jretai.2003.09.005

Booth-Butterfield, S., & Reger, B. (2004). The message changes belief and the rest is theory: the "1% or less" milk campaign and reasoned action. *Preventive Medicine, 39*(3), 581-588.

Borgmeier, I., & Westenhoefer, J. (2009). Impact of different food label formats on healthiness evaluation and food choice of consumers: a randomized-controlled study. *BMC Public Health, 9*(1), 184.

Borle, S., Boatwright, P., Kadane, J. B., Nunes, J. C., & Shmueli, G. (2005). The Effect of Product Assortment Changes on Customer Retention. *Marketing Science, 24*(4), 616-622. doi:http://mktsci.journal.informs.org/content/by/year

Bower, J. A., & Turner, L. (2001). Effect of liking, brand name and price on purchase intention for branded, own label and economy line crisp snack foods. *Journal of Sensory Studies, 16*(1), 95-115. doi:10.1111/j.1745-459X.2001.tb00292.x

Breugelmans, E., & Campo, K. (2011). Effectiveness of In-Store Displays in a Virtual Store Environment. *Journal of Retailing, 87*(1), 75-89. doi:10.1016/j.jretai.2010.09.003

Briesch, R. A., Chintagunta, P. K., & Fox, E. J. (2009). How does assortment affect grocery store choice? *Journal of Marketing research, 46*(2), 176-189.

Briesch, R. A., Dillon, W. R., & Fox, E. J. (2013). Category Positioning and Store Choice: The Role of Destination Categories. *Marketing Science, 32*(3), 488-509. doi:http://mktsci.journal.informs.org/content/by/year

Brissette, I., Lowenfels, A., Noble, C., & Spicer, D. (2013). Predictors of total calories purchased at fast-food restaurants: restaurant characteristics, calorie awareness, and use of calorie information. *Journal of Nutrition Education and Behavior, 45*(5), 404-411.

Broniarczyk, S. M., Hoyer, W. D., & McAlister, L. (1998). Consumers' perceptions of the assortment offered in a grocery category: The impact of item reduction. *Journal of marketing research*, 166-176.

Bronnenberg, B. J., Mela, C. F., & Boulding, W. (2006). The periodicity of pricing. *Journal of Marketing Research, 43*(3), 477-493.

Brown, D. M., & Tammineni, S. K. (2009). Managing sales of beverages in schools to preserve profits and improve children's nutrition intake in 15 Mississippi schools. *Journal of the American Dietetic Association, 109*(12), 2036-2042.

Brownbill, A. L., Miller, C. L., & Braunack-Mayer, A. J. (2018). Industry use of 'better-for-you' features on labels of sugar-containing beverages. *Public health nutrition, 21*(18), 3335-3343. doi:10.1017/s1368980018002392

Budd, N., Jeffries, J. K., Jones-Smith, J., Kharmats, A., McDermott, A. Y., & Gittelsohn, J. (2017). Store-directed price promotions and communications strategies improve healthier food supply and demand: impact results from a randomized controlled, Baltimore City store-intervention trial. *Public health nutrition, 20*(18), 3349-3359.

Buscher, L. A., Martin, K. A., & Crocker, S. (2001). Point-of-purchase messages framed in terms of cost, convenience, taste, and energy improve healthful snack selection in a college foodservice setting. *Journal of the american dietetic association, 101*(8), 909-913.

Bustillos, B., Sharkey, J. R., Anding, J., & McIntosh, A. (2009). Availability of More Healthful Food Alternatives in Traditional, Convenience, and Nontraditional Types of Food Stores in Two Rural Texas Counties. *Journal of the american dietetic association, 109*(5), 883-889. doi:10.1016/j.jada.2009.02.011

Butler, J. M., & Vossler, C. A. (2018). What Is an Unregulated and Potentially Misleading Label Worth? The Case of 'Natural'-Labelled Groceries. *Environmental and Resource Economics, 70*(2), 545-564. Retrieved from https://login.ezproxy.hioa.no/login?url=http://search.ebscohost.com/login.aspx?direct=true&db=ecn&AN=1712108&site=ehost-live
10.1007/s10640-017-0132-9

Caldwell, E. M., Kobayashi, M. M., DuBow, W., & Wytinck, S. (2009). Perceived access to fruits and vegetables associated with increased consumption. *Public health nutrition, 12*(10), 1743-1750.

Campo, K., Gijsbrechts, E., Goossens, T., & Verhetsel, A. (2000). The impact of location factors on the attractiveness and optimal space shares of product categories. *International Journal of Research in Marketing, 17*(4), 255-279.

Carroll, K. A., Bernard, J. C., & Pesek Jr, J. D. (2013). Consumer preferences for tomatoes: The influence of local, organic, and state program promotions by purchasing venue. *Journal of Agricultural and Resource Economics, 38*(3), 379-396. Retrieved from https://www.scopus.com/inward/record.uri?eid=2-s2.0-84894262238&partnerID=40&md5=bc1777b1fbfbbdb8e20a1162b08b57c2

Carroll, K. A., Samek, A., & Zepeda, L. (2018). Food bundling as a health nudge: investigating consumer fruit and vegetable selection using behavioral economics. *Appetite, 121*, 237-248. doi:10.1016/j.appet.2017.11.082

Chandon, P., Hutchinson, J. W., Bradlow, E. T., & Young, S. H. (2009). Does in-store marketing work? Effects of the number and position of shelf facings on brand attention and evaluation at the point of purchase. *Journal of Marketing, 73*(6), 1-17.

Chandon, P., & Wansink, B. (2002). When are stockpiled products consumed faster? A convenience–salience framework of postpurchase consumption incidence and quantity. *Journal of Marketing research, 39*(3), 321-335.

Cheadle, A., Psaty, B. M., Curry, S., Wagner, E., Diehr, P., Koepsell, T., & Kristal, A. (1991). Community-level comparisons between the grocery store environment and individual dietary practices. *Preventive Medicine, 20*(2), 250-261.

Cheadle, A., Psaty, B. M., Curry, S., Wagner, E., Diehr, P., Koepsell, T., & Kristal, A. (1993). Can measures of the grocery store environment be used to track community-level dietary changes? *Preventive Medicine, 22*(3), 361-372.

Cheadle, A., Psaty, B. M., Diehr, P., Koepsell, T., Wagner, E., Curry, S., & Kristal, A. (1995). Evaluating community-based nutrition-programs: Comparing grocery store and individual-level survey measures of program impact. *Preventive Medicine, 24*(1), 71-79.

Chintakayala, P. K., Young, W., Barkemeyer, R., & Morris, M. A. (2018). Breaking niche sustainable products into the mainstream: Organic milk and free-range eggs. *Business Strategy and the Environment, 27*(7), 1039-1051. doi:10.1002/bse.2050

Chu, J., Chintagunta, P., & Cebollada, J. (2008). A Comparison of Within-Household Price Sensitivity across Online and Offline Channels. *Marketing Science, 27*(2), 283-299. doi:http://mktsci.journal.informs.org/content/by/year

Chu, Y. H., Frongillo, E. A., Jones, S. J., & Kaye, G. L. (2009). Improving patrons' meal selections through the use of point-of-selection nutrition labels. *American journal of public health, 99*(11), 2001-2005.

Cinciripini, P. M. (1984). Changing food selections in a public cafeteria an applied behavior analysis. *Behavior Modification, 8*(4), 520-539.

Clement, J., Aastrup, J., & Charlotte Forsberg, S. (2015). Decisive visual saliency and consumers' in-store decisions. *Journal of Retailing and Consumer Services, 22*, 187-194. doi:10.1016/j.jretconser.2014.09.002

Coffino, J. A., Udo, T., & Hormes, J. M. (2020). Nudging while online grocery shopping: A randomized feasibility trial to enhance nutrition in individuals with food insecurity. *Appetite*, 104714. doi:https://dx.doi.org/10.1016/j.appet.2020.104714

Cohen, D. A., & Babey, S. H. (2012). Contextual influences on eating behaviours: Heuristic processing and dietary choices. *Obesity reviews, 13*(9), 766-779. doi:10.1111/j.1467-789X.2012.01001.x

Cohen, D. A., Bogart, L., Castro, G., Rossi, A. D., Williamson, S., & Han, B. (2018). Beverage marketing in retail outlets and the balance calories initiative. *Preventive medicine, 115*, 1-7. doi:10.1016/j.ypmed.2018.07.014

Cohen, D. A., Collins, R., Hunter, G., Ghosh-Dastidar, B., & Dubowitz, T. (2015). Store Impulse Marketing Strategies and Body Mass Index. *American journal of public health, 105*(7), 1446-1452. doi:10.2105/ajph.2014.302220

Cohen, J. F. W., Richardson, S. A., Cluggish, S. A., Parker, E., Catalano, P. J., & Rimm, E. B. (2015). Effects of choice architecture and chef-enhanced meals on the selection and consumption of healthier school foods: a randomized clinical trial. *JAMA Pediatrics, 169*(5), 431-437.

Colapinto, C. K., & Malaviarachchi, D. (2009). Paint your plate: effectiveness of a point-of-purchase display. *Canadian Journal of Dietetic Practice and Research, 70*(2), 66-71.

Cotugna, N., & Vickery, C. E. (1992). Development and supermarket field testing of videotaped nutrition messages for cancer risk reduction. *Public Health Reports, 107*(6), 691-694. Retrieved from https://www.scopus.com/inward/record.uri?eid=2-s2.0-0026472018&partnerID=40&md5=118f7c32ab01566a2c9d382b7b960c2f

Coucke, N., Vermeir, I., Slabbinck, H., & Van Kerckhove, A. (2019). Show me more! The influence of visibility on sustainable food choices. *Foods, 8*(6). doi:10.3390/foods8060186

Crawford, S. M., & Kalina, L. (1993). The shop smart tour. *Journal of Nutrition Education, 25*(2), 100B.

Crockett, R. A., Jebb, S. A., Hankins, M., & Marteau, T. M. (2014). The impact of nutritional labels and socioeconomic status on energy intake. An experimental field study. *Appetite, 81*, 12-19.

Crockett, R. A., King, S. E., Marteau, T. M., Prevost, A. T., Bignardi, G., Roberts, N. W., . . . Jebb, S. A. (2018). Nutritional labelling for healthier food or non-alcoholic drink purchasing and consumption. *The Cochrane database of systematic reviews, 2*, CD009315. doi:https://dx.doi.org/10.1002/14651858.CD009315.pub2

Cucchiara, C., Kwon, S., & Ha, S. J. (2015). Message framing and consumer responses to organic seafood labeling. *British Food Journal, 117*(5), 1547-1563. doi:10.1108/bfj-07-2014-0261

Cui, T. H., Raju, J. S., & Zhang, Z. J. (2008). A price discrimination model of trade promotions. *Marketing Science, 27*(5), 779-795.

Cummins, S., & Macintyre, S. (2006). Food environments and obesity—neighbourhood or nation? *International journal of epidemiology, 35*(1), 100-104.

Curhan, R. C. (1974). The effects of merchandising and temporary promotional activities on the sales of fresh fruits and vegetables in supermarkets. *Journal of Marketing Research, 11*(3), 286-294.

Curran, S., Gittelsohn, J., Anliker, J., Ethelbah, B., Blake, K., Sharma, S., & Caballero, B. (2005). Process evaluation of a store-based environmental obesity intervention on two American Indian Reservations. *Health Education Research, 20*(6), 719-729.

Daliani, D., Stott, S., Fiore, A., & Cottin, S. (2019). The effect of the enrichment of bread with fibres from fava bean husk by-products on postprandial satiety response and palatability. *Proceedings of the Nutrition Society, 78*(OCE2). doi:10.1017/S0029665119000079

Dannefer, R., Williams, D. A., Baronberg, S., & Silver, L. (2012). Healthy bodegas: increasing and promoting healthy foods at corner stores in New York City. *American journal of public health, 102*(10), e27-e31.

Dawson, J. (2013). Retailer activity in shaping food choice. *Food Quality and Preference, 28*(1), 339-347. doi:10.1016/j.foodqual.2012.09.012

Dayan, E., & Bar-Hillel, M. (2011). Nudge to nobesity II: Menu positions influence food orders. *Judgment and Decision making, 6*(4), 333-342.

De Bondt, C., Van Kerckhove, A., & Geuens, M. (2017). 'My lips are sealed'-The impact of package resealability on the consumption of tempting foods. *Appetite, 117*, 143-151.

De Wijk, R. A., Maaskant, A. J., Polet, I. A., Holthuysen, N. T. E., Kleef, E. V., & Vingerhoeds, M. H. (2016). An in-store experiment on the effect of accessibility on sales of wholegrain and white bread in supermarkets. *PLoS ONE, 11*(3). doi:10.1371/journal.pone.0151915

Degeratu, A. M., Rangaswamy, A., & Wu, J. (2000). Consumer choice behavior in online and traditional supermarkets: The effects of brand name, price, and other search attributes. *International Journal of Research in Marketing, 17*(1), 55-78. Retrieved from https://www.scopus.com/inward/record.uri?eid=2-s2.0-0034147928&partnerID=40&md5=aba5c5600320470fe0b0fa9aee2bad63

Demarque, C., Charalambides, L., Hilton, D. J., & Waroquier, L. (2015). Nudging sustainable consumption: The use of descriptive norms to promote a minority behavior in a realistic online shopping environment. *Journal of Environmental Psychology, 43*, 166-174. doi:10.1016/j.jenvp.2015.06.008

Desai, K. K., & Ratneshwar, S. (2003). Consumer perceptions of product variants positioned on atypical attributes. *Journal of the Academy of Marketing Science, 31*(1), 22-35.

Desai, K. K., & Talukdar, D. (2003). Relationship between product groups' price perceptions, shopper's basket size, and grocery store's overall store price image. *Psychology & Marketing, 20*(10), 903-933.

Desai, P. S. (2000). Multiple Messages to Retain Retailers: Signaling New Product Demand. *Marketing Science, 19*(4), 381-389. doi:http://mktsci.journal.informs.org/content/by/year

Desmet, P., & Renaudin, V. (1998). Estimation of product category sales responsiveness to allocated shelf space. *International Journal of Research in Marketing, 15*(5), 443-457.

Dhar, R., & Wertenbroch, K. (2000). Consumer choice between hedonic and utilitarian goods. *Journal of Marketing Research, 37*(1), 60-71.

Dhar, S. K., & Hoch, S. J. (1996). Price discrimination using in-store merchandising. *The Journal of Marketing*, 17-30.

Dhar, S. K., Hoch, S. J., & Kumar, N. (2001). Effective category management depends on the role of the category☆. *Journal of Retailing, 77*(2), 165-184.

Diliberti, N., Bordi, P. L., Conklin, M. T., Roe, L. S., & Rolls, B. J. (2004). Increased portion size leads to increased energy intake in a restaurant meal. *Obesity research, 12*(3), 562-568.

DiSantis, K. I., Birch, L. L., Davey, A., Serrano, E. L., Zhang, J., Bruton, Y., & Fisher, J. O. (2013). Plate size and children's appetite: effects of larger dishware on self-served portions and intake. *Pediatrics, 131*(5), e1451-e1458.

Do Vale, R. C., Pieters, R., & Zeelenberg, M. (2008). Flying under the radar: Perverse package size effects on consumption self-regulation. *Journal of Consumer Research, 35*(3), 380-390.

Doble, B., Ang Jia Ler, F., & Finkelstein, E. A. (2020). The effect of implicit and explicit taxes on the purchasing of 'high-in-calorie' products: A randomized controlled trial. *Economics and Human Biology, 37*, 100860. doi:https://dx.doi.org/10.1016/j.ehb.2020.100860

Donnelly, G. E., Zatz, L. Y., Svirsky, D., & John, L. K. (2018). The effect of graphic warnings on sugary-drink purchasing. *Psychological Science, 29*(8), 1321-1333.

Dougherty, M.-F., Wittsten, A. B., & Guarino, M. A. (1990). Promoting low-fat foods in the supermarket using various methods, including videocassettes. *Journal of the american dietetic association, 90*(8), 1106-1108.

Downs, J. S., Wisdom, J., Wansink, B., & Loewenstein, G. (2013). Supplementing menu labeling with calorie recommendations to test for facilitation effects. *American journal of public health, 103*(9), 1604-1609.

Drèze, X., & Hoch, S. J. (1998). Exploiting the installed base using cross-merchandising and category destination programs. *International Journal of Research in Marketing, 15*(5), 459-471.

Drichoutis, A. C., Lazaridis, P., & Nayga Jr, R. M. (2007). An assessment of product class involvement in food-purchasing behavior. *European Journal of Marketing, 41*(7-8), 888-914. doi:10.1108/03090560710752447

Drichoutis, A. C., Lazaridis, P., & Nayga, R. M. (2005). Nutrition Knowledge and Consumer Use of Nutritional Food Labels. *European Review of Agricultural Economics, 32*(1), 93-118. doi:https://academic.oup.com/erae/issue

Drichoutis, A. C., Lazaridis, P., & Nayga, R. M. (2009). Would consumers value food-away-from-home products with nutritional labels? *Agribusiness, 25*(4), 550-575.

Dubbert, P. M., Johnson, W. G., Schlundt, D. G., & Montague, N. W. (1984). The influence of caloric information on cafeteria food choices. *Journal of applied behavior analysis, 17*(1), 85-92.

Ducrot, P., Julia, C., Mejean, C., Kesse-Guyot, E., Touvier, M., Fezeu, L. K., . . . Peneau, S. (2016). Impact of Different Front-of-Pack Nutrition Labels on Consumer Purchasing Intentions: A Randomized Controlled Trial. *Am J Prev Med, 50*(5), 627-636. doi:10.1016/j.amepre.2015.10.020

Duffett, R. G., & Foster, C. (2017). Shopping list development and use of advertisements' pre-store food-buying practices within different socio-economic status areas in South Africa. *British Food Journal, 119*(12), 2880-2902. doi:10.1108/BFJ-11-2016-0556

Duffey, K. J., Gordon-Larsen, P., Shikany, J. M., Guilkey, D., Jacobs, D. R., & Popkin, B. M. (2010). Food price and diet and health outcomes: 20 years of the CARDIA Study. *Archives of internal medicine, 170*(5), 420-426.

Dulsrud, A., & Jacobsen, E. (2009). In-Store Marketing as a Mode of Discipline. *Journal of Consumer Policy, 32*(3), 203-218. doi:https://link.springer.com/journal/volumesAndIssues/10603

Dumanovsky, T., Huang, C. Y., Nonas, C. A., Matte, T. D., Bassett, M. T., & Silver, L. D. (2011). Changes in energy content of lunchtime purchases from fast food restaurants after introduction of calorie labelling: cross sectional customer surveys. *Bmj, 343*.

Duncan Herrington, J. (1996). Effects of music in service environments: A field study. *Journal of Services Marketing, 10*(2), 26-41. doi:10.1108/08876049610114249

Dunn, R. A., Dean, W. R., Johnson, C. M., Leidner, A., & Sharkey, J. R. (2012). The Effect of Distance and Cost on Fruit and Vegetable Consumption in Rural Texas. *Journal of Agricultural and Applied Economics, 44*(4), 491-500. doi:http://journals.cambridge.org/action/displayBackIssues?jid=AAE

Dwivedi, G., Harvey, J., & Close, A. (1997). Taste-test booth: an innovative tool in health promotion. *Journal of the Canadian Dietetic Association*.

Ebster, C., Wagner, U., & Neumueller, D. (2009). Children's influences on in-store purchases. *Journal of Retailing and Consumer Services, 16*(2), 145-154.

Edge MS. (2010). *International Food Information Council Foundation Front-of-Pack Labeling Consumer Research Project. Paper presented at: Workshop on Consumer Behavior Research and Front-of-Package Nutrition Rating Systems and Symbols: What Do Consumers Know, Understand, and Use?* Retrieved from

Elbel, B., Gyamfi, J., & Kersh, R. (2011). Child and adolescent fast-food choice and the influence of calorie labeling: a natural experiment. *International journal of obesity, 35*(4), 493-500.

Elbel, B., Kersh, R., Brescoll, V. L., & Dixon, L. B. (2009). Calorie Labeling And Food Choices: A First Look At The Effects On Low-Income People In New York City: Calorie information on menus appears to increase awareness of calorie content, but not necessarily the number of calories people purchase. *Health Affairs, 28*(Suppl1), w1110-w1121.

Elbel, B., Mijanovich, T., Dixon, L. B., Abrams, C., Weitzman, B., Kersh, R., . . . Ogedegbe, G. (2013). Calorie labeling, fast food purchasing and restaurant visits. *Obesity, 21*(11), 2172-2179.

Elberg, A., Gardete, P. M., Macera, R., & Noton, C. (2019). Dynamic Effects of Price Promotions: Field Evidence, Consumer Search, and Supply-Side Implications. *Quantitative Marketing and Economics, 17*(1), 1-58. Retrieved from https://login.ezproxy.hioa.no/login?url=http://search.ebscohost.com/login.aspx?direct=true&db=ecn&AN=1758367&site=ehost-live
10.1007/s11129-018-9205-5

Elliott, C. (2008). Marketing fun foods: a profile and analysis of supermarket food messages targeted at children. *Canadian Public Policy, 34*(2), 259-273.

Elliott, C. D. (2009). Healthy food looks serious: How children interpret packaged food products. *Canadian Journal of Communication, 34*(3), 359.

Ellison, B., Lusk, J. L., & Davis, D. (2013). Looking at the label and beyond: the effects of calorie labels, health consciousness, and demographics on caloric intake in restaurants. *International journal of behavioral nutrition and physical activity, 10*(1), 21.

Elofsson, K., Bengtsson, N., Matsdotter, E., & Arntyr, J. (2016). The impact of climate information on milk demand: Evidence from a field experiment. *Food Policy, 58*, 14-23.

Elsbernd, S. L., Reicks, M. M., Mann, T. L., Redden, J. P., Mykerezi, E., & Vickers, Z. M. (2016). Serving vegetables first: A strategy to increase vegetable consumption in elementary school cafeterias. *Appetite, 96*, 111-115.

Ensaff, H., Homer, M., Sahota, P., Braybrook, D., Coan, S., & McLeod, H. (2015). Food choice architecture: An intervention in a secondary school and its impact on students' plant-based food choices. *Nutrients, 7*(6), 4426-4437.

Epstein, L. H., Dearing, K. K., Handley, E. A., Roemmich, J. N., & Paluch, R. A. (2006). Relationship of mother and child food purchases as a function of price: a pilot study. *Appetite, 47*(1), 115-118.

Epstein, L. H., Dearing, K. K., Paluch, R. A., Roemmich, J. N., & Cho, D. (2007). Price and maternal obesity influence purchasing of low-and high-energy-dense foods. *The American Journal of Clinical Nutrition, 86*(4), 914-922.

Epstein, L. H., Dearing, K. K., Roba, L. G., & Finkelstein, E. (2010). The influence of taxes and subsidies on energy purchased in an experimental purchasing study. *Psychological Science, 21*(3), 406-414.

Epstein, L. H., Finkelstein, E., Raynor, H., Nederkoorn, C., Fletcher, K. D., Jankowiak, N., & Paluch, R. A. (2015). Experimental analysis of the effect of taxes and subsides on calories purchased in an on-line supermarket. *Appetite, 95*, 245-251.

Epstein, L. H., Finkelstein, E. A., Katz, D. L., Jankowiak, N., Pudlewski, C., & Paluch, R. A. (2016). Effects of nutrient profiling and price changes based on NuVal® scores on food purchasing in an online experimental supermarket. *Public health nutrition, 19*(12), 2157-2164.

Epstein, L. H., Handley, E. A., Dearing, K. K., Cho, D. D., Roemmich, J. N., Paluch, R. A., . . . Spring, B. (2006). Purchases of food in youth: influence of price and income. *Psychological Science, 17*(1), 82-89.

Escaron, A. L., Meinen, A. M., Nitzke, S. A., & Martinez-Donate, A. P. (2013). Supermarket and grocery store-based interventions to promote healthful food choices and eating practices: A systematic review. *Preventing chronic disease, 10*(4), 1-20. doi:10.5888/pcd10.120156

Feunekes, G. I., Gortemaker, I. A., Willems, A. A., Lion, R., & Van Den Kommer, M. (2008). Front-of-pack nutrition labelling: testing effectiveness of different nutrition labelling formats front-of-pack in four European countries. *Appetite, 50*(1), 57-70.

Finkelstein, E. A., Strombotne, K. L., Chan, N. L., & Krieger, J. (2011). Mandatory menu labeling in one fast-food chain in King County, Washington. *American journal of preventive medicine, 40*(2), 122-127.

Finnell, K. J., John, R., & Thompson, D. M. (2017). 1% low-fat milk has perks!: An evaluation of a social marketing intervention. *Preventive Medicine Reports, 5*, 144-149. doi:10.1016/j.pmedr.2016.11.017

Fletcher, J. M., Frisvold, D., & Tefft, N. (2010). Can soft drink taxes reduce population weight? *Contemporary Economic Policy, 28*(1), 23-35.

Foerster, S. B., Gregson, J., Beall, D. L., Hudes, M., Magnuson, H., Livingston, S., . . . Garbolino, T. (1998). The California Children's 5 a Day-Power Play! Campaign: Evaluation of a Large-Scale Social Marketing Initiative. *Family & Community Health, 21*(1), 46-64.

Foerster, S. B., Kizer, K. W., DiSogra, L. K., Bal, D. G., Krieg, B. F., & Bunch, K. L. (1995). California's "5 a day—for better health!" campaign: an innovative population-based effort to effect large-scale dietary change. *American journal of preventive medicine, 11*(2), 124-131.

Forwood, S. E., Ahern, A. L., Marteau, T. M., & Jebb, S. A. (2015). Offering within-category food swaps to reduce energy density of food purchases: a study using an experimental online supermarket. *International journal of behavioral nutrition and physical activity, 12*(1), 1-10.

Franco, M., Diez-Roux, A. V., Nettleton, J. A., Lazo, M., Brancati, F., Caballero, B., . . . Moore, L. V. (2009). Availability of healthy foods and dietary patterns: the Multi-Ethnic Study of Atherosclerosis. *The American Journal of Clinical Nutrition, 89*(3), 897-904.

Freedman, D. A., Bell, B. A., & Collins, L. V. (2011). The Veggie Project: a case study of a multi-component farmers' market intervention. *The journal of primary prevention, 32*(3-4), 213.

Freedman, M. R., & Brochado, C. (2010). Reducing portion size reduces food intake and plate waste. *Obesity, 18*(9), 1864-1866.

Freedman, M. R., & Connors, R. (2010). Point-of-purchase nutrition information influences food-purchasing behaviors of college students: a pilot study. *Journal of the american dietetic association, 110*(8), 1222-1226.

Freedman, M. R., & Larner, J. M. (2011). Point-of-selection nutrition information influences choice of portion size in an all-you-can-eat university dining hall. *Journal of Foodservice Business Research, 14*(1), 86-98.

Freire, T., & Rudkin, S. (2019). Healthy Food Diversity and Supermarket Interventions: Evidence from the Seacroft Intervention Study. *Food Policy, 83*, 125-138. Retrieved from https://login.ezproxy.hioa.no/login?url=http://search.ebscohost.com/login.aspx?direct=true&db=ecn&AN=1762672&site=ehost-live

10.1016/j.foodpol.2018.12.006

French, S. A., Harnack, L. J., Hannan, P. J., Mitchell, N. R., Gerlach, A. F., & Toomey, T. L. (2010). Worksite environment intervention to prevent obesity among metropolitan transit workers. *Preventive medicine, 50*(4), 180-185.

French, S. A., Jeffery, R. t., Story, M., Breitlow, K. K., Baxter, J. S., Hannan, P., & Snyder, M. P. (2001). Pricing and promotion effects on low-fat vending snack purchases: the CHIPS Study. *American journal of public health, 91*(1), 112.

French, S. A., Jeffery, R. W., Story, M., Hannan, P., & Snyder, M. P. (1997). A pricing strategy to promote low-fat snack choices through vending machines. *American journal of public health, 87*(5), 849-851.

French, S. A., Story, M., Jeffery, R. W., Snyder, P., Eisenberg, M., Sidebottom, A., & Murray, D. (1997). Pricing strategy to promote fruit and vegetable purchase in high school cafeterias. *Journal of the American Dietetic Association, 97*(9), 1008-1010.

Friis, R., Skov, L. R., Olsen, A., Appleton, K. M., Saulais, L., Dinnella, C., . . . Giboreau, A. (2017). Comparison of three nudge interventions (priming, default option, and perceived variety) to promote vegetable consumption in a self-service buffet setting. *PLoS ONE, 12*(5), e0176028.

Gaigi, H., Raffin, S., Maillot, M., Adrover, L., Ruffieux, B., & Darmon, N. (2015). Expérimentation d'un fléchage nutritionnel dans deux supermarchés à Marseille «Le choix Vita+». *Cahiers de Nutrition et de Dietetique, 50*(1), 16-24.

Geaney, F., Kelly, C., Di Marrazzo, J. S., Harrington, J. M., Fitzgerald, A. P., Greiner, B. A., & Perry, I. J. (2016). The effect of complex workplace dietary interventions on employees' dietary intakes, nutrition knowledge and health status: a cluster controlled trial. *Preventive medicine, 89*, 76-83.

Giesen, J. C., Havermans, R. C., Nederkoorn, C., & Jansen, A. (2012). Impulsivity in the supermarket. Responses to calorie taxes and subsidies in healthy weight undergraduates. *Appetite, 58*(1), 6-10. doi:https://doi.org/10.1016/j.appet.2011.09.026

Giesen, J. C., Payne, C. R., Havermans, R. C., & Jansen, A. (2011). Exploring how calorie information and taxes on high-calorie foods influence lunch decisions. *The American journal of clinical nutrition, 93*(4), 689-694.

Gill, L., & Rudkin, S. (2014). Deconstructing Supermarket Intervention Effects on Fruit and Vegetable Consumption in Areas of Limited Retail Access: Evidence from the Seacroft Study. *Environment and Planning A, 46*(3), 649-665. doi:http://epn.sagepub.com/content/by/year

Gittelsohn, J., Anliker, J. A., Sharma, S., Vastine, A. E., Caballero, B., & Ethelbah, B. (2006). Psychosocial determinants of food purchasing and preparation in American Indian households. *Journal of Nutrition Education and Behavior, 38*(3), 163-168.

Gittelsohn, J., Dyckman, W., Tan, M. L., Boggs, M. K., Frick, K. D., Alfred, J., . . . Palafox, N. A. (2006). Development and implementation of a food store–based intervention to improve diet in the Republic of the Marshall Islands. *Health promotion practice, 7*(4), 396-405.

Gittelsohn, J., Kim, E. M., He, S., & Pardilla, M. (2013). A food store–based environmental intervention is associated with reduced BMI and improved psychosocial factors and food-related behaviors on the Navajo Nation. *The Journal of nutrition, 143*(9), 1494-1500.

Gittelsohn, J., Song, H.-J., Suratkar, S., Kumar, M. B., Henry, E. G., Sharma, S., . . . Anliker, J. A. (2010). An urban food store intervention positively affects food-related psychosocial variables and food behaviors. *Health Education & Behavior, 37*(3), 390-402.

Gittelsohn, J., Suratkar, S. i., Song, H.-J., Sacher, S., Rajan, R., Rasooly, I. R., . . . Anliker, J. A. (2010). Process evaluation of Baltimore Healthy Stores: a pilot health intervention program with supermarkets and corner stores in Baltimore City. *Health promotion practice, 11*(5), 723-732.

Gittelsohn, J., Vijayadeva, V., Davison, N., Ramirez, V., Cheung, L. W. K., Murphy, S., & Novotny, R. (2010). A food store intervention trial improves caregiver psychosocial factors and children's dietary intake in Hawaii. *Obesity, 18*(S1), S84-S90.

Glanz, K., & Yaroch, A. L. (2004). Strategies for increasing fruit and vegetable intake in grocery stores and communities: Policy, pricing, and environmental change. *Preventive medicine, 39*(SUPPL. 2), 75-80. doi:10.1016/j.ypmed.2004.01.004

Gopalan, A., Shaw, P. A., Lim, R., Paramanund, J., Patel, D., Zhu, J., . . . Buttenheim, A. M. (2019). Use of financial incentives and text message feedback to increase healthy food purchases in a grocery store cash back program: a randomized controlled trial. *BMC public health, 19*(1), 674. doi:10.1186/s12889-019-6936-5

Gordon-Larsen, P., Guilkey, D. K., & Popkin, B. M. (2011). An economic analysis of community-level fast food prices and individual-level fast food intake: a longitudinal study. *Health & place, 17*(6), 1235-1241.

Gorton, D., Mhurchu, C. N., Chen, M.-h., & Dixon, R. (2009). Nutrition labels: a survey of use, understanding and preferences among ethnically diverse shoppers in New Zealand. *Public health nutrition, 12*(09), 1359-1365.

Goto, K., Waite, A., Wolff, C., Chan, K., & Giovanni, M. (2013). Do environmental interventions impact elementary school students' lunchtime milk selection? *Applied Economic Perspectives and Policy, 35*(2), 360-376.

Green, R. (2006). Green for go? *Food Manuf., 81*, 48-48.

Greene, K. N., Gabrielyan, G., Just, D. R., & Wansink, B. (2017). Fruit-promoting smarter lunchrooms interventions: results from a cluster RCT. *American journal of preventive medicine, 52*(4), 451-458.

Grunert, K. G., Fernández-Celemín, L., Wills, J. M., genannt Bonsmann, S. S., & Nureeva, L. (2010a). Use and understanding of nutrition information on food labels in six European countries. *Journal of Public Health, 18*(3), 261-277.

Grunert, K. G., Wills, J. M., & Fernández-Celemín, L. (2010b). Nutrition knowledge, and use and understanding of nutrition information on food labels among consumers in the UK. *Appetite, 55*(2), 177-189.

Guan, X., Atlas, S. A., & Vadiveloo, M. (2018). Targeted retail coupons influence category-level food purchases over 2-years 11 Medical and Health Sciences 1117 Public Health and Health Services. *International journal of behavioral nutrition and physical activity, 15*(1). doi:10.1186/s12966-018-0744-7

Guiding Stars Licensing Company. (2010a). Response to Docket FDA-2010-N-0210. Comment 3. Retrieved from http://www.regulations.gov/#!documentDetail;D
=FDA-2010-N-0210-0090

Guiding Stars Licensing Company. (2010b). Response to Docket FDA-2010-N-0210. Comment 3. 2010. . In *19 November 2012*.

Hamlin, R., & McNeill, L. (2016). Does the Australasian "Health Star Rating" Front of Pack Nutritional Label System Work? *Nutrients, 8*(6), 327.

Hanks, A. S., Just, D. R., Smith, L. E., & Wansink, B. (2012). Healthy convenience: nudging students toward healthier choices in the lunchroom. *Journal of Public Health, 34*(3), 370-376.

Hanks, A. S., Just, D. R., & Wansink, B. (2013). Smarter lunchrooms can address new school lunchroom guidelines and childhood obesity. *The Journal of pediatrics, 162*(4), 867-869.

Harnack, L. J., & French, S. A. (2008). Effect of point-of-purchase calorie labeling on restaurant and cafeteria food choices: a review of the literature. *International Journal of Behavioral Nutrition and Physical Activity, 5*(1), 51.

Harris, J. L., Schwartz, M. B., & Brownell, K. D. (2010). Marketing foods to children and adolescents: licensed characters and other promotions on packaged foods in the supermarket. *Public health nutrition, 13*(03), 409-417.

Harris, J. L., Schwartz, M. B., & Kelly, B. D. (2009). *Cereal f.a.c.t.s: nutrition and marketing ratings of children's cereals*. Retrieved from New Haven CT, Yale University, Rudd Center for Food Policy and Obesity: https://www.cerealfacts.org/media/Cereal_FACTS_Report_Summary_2009.pdf

Hartmann-Boyce, J., Bianchi, F., Piernas, C., Payne Riches, S., Frie, K., Nourse, R., & Jebb, S. A. (2018). Grocery store interventions to change food purchasing behaviors: a systematic review of randomized controlled trials. *The American journal of clinical nutrition, 107*(6), 1004-1016. doi:https://dx.doi.org/10.1093/ajcn/nqy045

Hawkes, C. (2008). Dietary implications of supermarket development: a global perspective. *Development Policy Review, 26*(6), 657-692.

Hawkes, C. (2010). Food packaging: the medium is the message. *Public health nutrition, 13*(02), 297-299.

Healthy Corner Stores Network. (2009). *Healthy corner stores: the state of the movement 2009*. Retrieved from n.a.: http://www.changelabsolutions.org/sites/default/files/documents/HCSReport.pdf

Heilman, C. M., Nakamoto, K., & Rao, A. G. (2002). Pleasant surprises: Consumer response to unexpected in-store coupons. *Journal of Marketing Research, 39*(2), 242-252.

Herman, D. R., Harrison, G. G., Afifi, A. A., & Jenks, E. (2008). Effect of a targeted subsidy on intake of fruits and vegetables among low-income women in the special supplemental nutrition program for women, infants, and children. *American journal of public health, 98*(1), 98-105. doi:10.2105/ajph.2005.079418

Herman, D. R., Harrison, G. G., & Jenks, E. (2006). Choices made by low-income women provided with an economic supplement for fresh fruit and vegetable purchase. *Journal of the American Dietetic Association, 106*(5), 740-744.

Ho, L. S., Gittelsohn, J., Rimal, R., Treuth, M. S., Sharma, S., Rosecrans, A., & Harris, S. B. (2008). An integrated multi-institutional diabetes prevention program improves knowledge and healthy food acquisition in northwestern Ontario First Nations. *Health Education & Behavior, 35*(4), 561-573.

Hoch, S. J., Bradlow, E. T., & Wansink, B. (1999). The variety of an assortment. *Marketing Science, 18*(4), 527-546.

Hoefkens, C., Lachat, C., Kolsteren, P., Van Camp, J., & Verbeke, W. (2011). Posting point-of-purchase nutrition information in university canteens does not influence meal choice and nutrient intake. *The American journal of clinical nutrition, 94*(2), 562-570.

Holweg, C., Schnedlitz, P., & Teller, C. (2009). The drivers of consumer value in the ECR Category Management model. *The International Review of Retail, Distribution and Consumer Research, 19*(3), 199-218.

Horgen, K. B., & Brownell, K. D. (2002). Comparison of price change and health message interventions in promoting healthy food choices. *Health Psychology, 21*(5), 505.

Hubbard, K. L., Bandini, L. G., Folta, S. C., Wansink, B., Eliasziw, M., & Must, A. (2015). Impact of a Smarter Lunchroom intervention on food selection and consumption among adolescents and young adults with intellectual and developmental disabilities in a residential school setting. *Public health nutrition, 18*(2), 361-371.

Huddleston, P., Whipple, J., Nye Mattick, R., & Jung Lee, S. (2009). Customer satisfaction in food retailing: comparing specialty and conventional grocery stores. *International Journal of Retail & Distribution Management, 37*(1), 63-80.

Hui, S. K., Bradlow, E. T., & Fader, P. S. (2009). Testing behavioral hypotheses using an integrated model of grocery store shopping path and purchase behavior. *Journal of Consumer Research, 36*(3), 478-493.

Huitink, M., Poelman, M. P., van den Eynde, E., Seidell, J. C., & Dijkstra, S. C. (2020). Social norm nudges in shopping trolleys to promote vegetable purchases: A quasi-experimental study in a supermarket in a deprived urban area in the Netherlands. *Appetite, 151*. doi:10.1016/j.appet.2020.104655

Hunt, M. K., Lefebvre, R. C., Hixson, M. L., Banspach, S., Assaf, A., & Carleton, R. (1990). Pawtucket Heart Health Program point-of-purchase nutrition education program in supermarkets. *American journal of public health, 80*(6), 730-732.

Hunter, B. T. (2002). Marketing foods to kids: Using fun to sell. *Consumers' Research Magazine, 85*(3), 16-19.

Inman, J. J., Winer, R. S., & Ferraro, R. (2009). The interplay among category characteristics, customer characteristics, and customer activities on in-store decision making. *Journal of Marketing, 73*(5), 19-29.

Iyengar, S. S., & Lepper, M. R. (2000). When choice is demotivating: Can one desire too much of a good thing? *Journal of personality and Social Psychology, 79*(6), 995.

Jeffery, R. W., French, S. A., Raether, C., & Baxter, J. E. (1994). An environmental intervention to increase fruit and salad purchases in a cafeteria. *Preventive Medicine, 23*(6), 788-792.

Jensen, J. M. (1995). Children's purchase requests and parental responses: results from an exploratory study in Denmark. *E-European Advances in Consumer Research Volume 2*.

Jones, G., & Richardson, M. (2007). An objective examination of consumer perception of nutrition information based on healthiness ratings and eye movements. *Public health nutrition, 10*(03), 238-244.

Jue, J. J. S., Press, M. J., McDonald, D., Volpp, K. G., Asch, D. A., Mitra, N., . . . Loewenstein, G. (2012). The impact of price discounts and calorie messaging on beverage consumption: a multi-site field study. *Preventive medicine, 55*(6), 629-633.

Julia, C., Blanchet, O., Méjean, C., Péneau, S., Ducrot, P., Allès, B., . . . et al. (2016). Impact of the front-of-pack 5-colour nutrition label (5-CNL) on the nutritional quality of purchases: an experimental study. *International journal of behavioral nutrition and physical activity, 13*(1), 101. doi:10.1186/s12966-016-0416-4

Kahn, B. E., & Wansink, B. (2004). The influence of assortment structure on perceived variety and consumption quantities. *Journal of Consumer Research, 30*(4), 519-533.

Kelly, B., Hughes, C., Chapman, K., Louie, J. C.-Y., Dixon, H., Crawford, J., . . . Slevin, T. (2009). Consumer testing of the acceptability and effectiveness of front-of-pack food labelling systems for the Australian grocery market. *Health Promotion International, 24*(2), 120-129.

Kennedy, B. M., Champagne, C. M., Ryan, D. H., Newton, J. R., Conish, B. K., Harsha, D. W., . . . Bogle, M. L. (2009). The" Rolling Store:" an economical and environmental approach to the prevention of weight gain in African American women. *Ethnicity & disease, 19*(1), 7-12.

Khan, T., Powell, L. M., & Wada, R. (2012). Fast food consumption and food prices: evidence from panel data on 5th and 8th grade children. *Journal of obesity, 2012*.

Kocken, P. L., Eeuwijk, J., Van Kesteren, N. M. C., Dusseldorp, E., Buijs, G., Bassa-Dafesh, Z., & Snel, J. (2012). Promoting the purchase of low-calorie foods from school vending machines: a cluster-randomized controlled study. *Journal of School Health, 82*(3), 115-122.

Kottke, T. E., Pronk, N. P., Katz, A. S., Tillema, J. O., & Flottemesch, T. J. (2013). Peer Reviewed: The Effect of Price Reduction on Salad Bar Purchases at a Corporate Cafeteria. *Preventing chronic disease, 10*.

Koutoukidis, D. A., Jebb, S. A., Ordonez-Mena, J. M., Noreik, M., Tsiountsioura, M., Kennedy, S., . . . Piernas, C. (2019). Prominent positioning and food swaps are effective interventions to reduce the saturated fat content of the shopping basket in an experimental online supermarket: a randomized controlled trial. *International journal of behavioral nutrition and physical activity, 16*(1). doi:10.1186/s12966-019-0810-9

Kozup, J. C., Creyer, E. H., & Burton, S. (2003). Making healthful food choices: the influence of health claims and nutrition information on consumers' evaluations of packaged food products and restaurant menu items. *Journal of Marketing, 67*(2), 19-34.

Kral, T. V. E., Bannon, A. L., & Moore, R. H. (2016). Effects of financial incentives for the purchase of healthy groceries on dietary intake and weight outcomes among older adults: a randomized pilot study. *Appetite, 100*, 110-117. doi:10.1016/j.appet.2016.02.022

Krieger, J. W., Chan, N. L., Saelens, B. E., Ta, M. L., Solet, D., & Fleming, D. W. (2013). Menu labeling regulations and calories purchased at chain restaurants. *American journal of preventive medicine, 44*(6), 595-604.

Krishna, A., & Zhang, Z. J. (1999). Short-or long-duration coupons: The effect of the expiration date on the profitability of coupon promotions. *Management Science, 45*(8), 1041-1056.

Krishnan, T. V., & Rao, R. C. (1995). Double couponing and retail pricing in a couponed product category. *Journal of Marketing Research*, 419-432.

Kroese, F. M., Marchiori, D. R., & de Ridder, D. T. D. (2016). Nudging healthy food choices: a field experiment at the train station. *Journal of Public Health, 38*(2), e133-e137.

Lachat, C. K., Verstraeten, R., De Meulenaer, B., Menten, J., Huybregts, L. F., Van Camp, J., . . . Kolsteren, P. W. (2009). Availability of free fruits and vegetables at canteen lunch improves lunch and daily nutritional profiles: a randomised controlled trial. *British journal of nutrition, 102*(07), 1030-1037.

Lang, J. E., Mercer, N., Tran, D., & Mosca, L. (2000). Use of a supermarket shelf-labeling program to educate a predominately minority community about foods that promote heart health. *Journal of the American Dietetic Association, 100*(7), 804-809.

Larson, N., & Story, M. (2009). A review of environmental influences on food choices. *Ann Behav Med, 38 Suppl 1*, S56-73. doi:10.1007/s12160-009-9120-9

Larson, N. I., Story, M. T., & Nelson, M. C. (2009). Neighborhood environments: disparities in access to healthy foods in the US. *American journal of preventive medicine, 36*(1), 74-81. e10.

Larson, R. (2005). Making category management more practical. *Journal of Food Distribution Research, 36*(1), 101-105.

Larson, R. (2006). Core principles for supermarket aisle management. *Journal of Food Distribution Research, 37*(1), 101.

Larsson, I., Lissner, L., & Wilhelmsen, L. (1999). TheGreen Keyhole'revisited: Nutritional knowledge may influence food selection. *European Journal of Clinical Nutrition, 52*, 776-780.

Le, H. N., Gold, L., Abbott, G., Crawford, D., McNaughton, S. A., Mhurchu, C. N., . . . Ball, K. (2016). Economic evaluation of price discounts and skill-building strategies on purchase and consumption of healthy food and beverages: the SHELf randomized controlled trial. *Social science & medicine, 159*, 83-91. doi:10.1016/j.socscimed.2016.04.015

Lemon, K. N., & Nowlis, S. M. (2002). Developing synergies between promotions and brands in different price–quality tiers. *Journal of Marketing Research, 39*(2), 171-185.

Lent, M. R., Vander Veur, S. S., McCoy, T. A., Wojtanowski, A. C., Sandoval, B., Sherman, S., . . . Foster, G. D. (2014). A randomized controlled study of a healthy corner store initiative on the purchases of urban, low-income youth. *Obesity, 22*(12), 2494-2500.

Levin, S. (1996). Pilot study of a cafeteria program relying primarily on symbols to promote healthy choices. *Journal of nutrition education, 28*(5), 282-285.

Levy, A. S., Mathews, O., Stephenson, M., Tenney, J. E., & Schucker, R. E. (1985). The impact of a nutrition information program on food purchases. *Journal of Public Policy & Marketing, 4*(1), 1-13.

Levy, A. S., Schucker, R. E., Tenney, J. E., & Mathews, O. (1988). Consumer demand for detailed nutrition information: a case study. *Journal of Nutrition Education, 20*(4), 161-166.

Levy, D. E., Riis, J., Sonnenberg, L. M., Barraclough, S. J., & Thorndike, A. N. (2012). Food choices of minority and low-income employees: a cafeteria intervention. *American journal of preventive medicine, 43*(3), 240-248.

Lichtenstein, D. R., Netemeyer, R. G., & Burton, S. (1995). Assessing the domain specificity of deal proneness: a field study. *Journal of Consumer Research, 22*(3), 314-326.

Light, L., Tenney, J., Portnoy, B., Kessler, L., Rodgers, A. B., Patterson, B., . . . Evans, S. K. (1989). Eat for health: a nutrition and cancer control supermarket intervention. *Public Health Reports, 104*(5), 443.

Lin C-TJ, & Levy A. (2010). *Food and Drug Administration's front-of-pack consumer research. Paper presented at: Workshop on Consumer Behavior Research and Front-of-Package Nutrition Rating Systems and Symbols: What Do Consumers Know, Understand, and Use?* Retrieved from Washington, DC.:

Lowe, M. R., Tappe, K. A., Butryn, M. L., Annunziato, R. A., Coletta, M. C., Ochner, C. N., & Rolls, B. J. (2010). An intervention study targeting energy and nutrient intake in worksite cafeterias. *Eating behaviors, 11*(3), 144-151.

Ma, Y., He, F. J., Li, N., Hao, J. J., Zhang, J., Yan, L. L., & Wu, Y. (2016). Salt sales survey: a simplified, cost-effective method to evaluate population salt reduction programs—a cluster-randomized trial. *Hypertension Research, 39*(4), 254-259.

Malam, S., Clegg, S., Kirwan, S., McGinigal, S., Raats, M., Shepherd, R., . . . Dean, M. (2009). Comprehension and use of UK nutrition signpost labelling schemes. *London: Food Standards Agency.*

Martínez-Donate, A. P., Riggall, A. J., Meinen, A. M., Malecki, K., Escaron, A. L., Hall, B., . . . Nitzke, S. (2015). Evaluation of a pilot healthy eating intervention in restaurants and food stores of a rural community: a randomized community trial. *BMC Public Health, 15*(1), 136.

Maubach, N., Hoek, J., & McCreanor, T. (2009b). An exploration of parents' food purchasing behaviours. *Appetite, 53*(3), 297-302. doi:https://doi.org/10.1016/j.appet.2009.07.005

Mayer, J. A., Heins, J. M., Vogel, J. M., Morrison, D. C., Lankester, L. D., & Jacobs, A. L. (1986). PROMOTING LOW-FAT ENTREE CHOICES IN A PUBLIC CAFETERIA. *Journal of Applied Behavior Analysis, 19*(4), 397-402.

Mazza, M. C., Dynan, L., Siegel, R. M., & Tucker, A. L. (2018). Nudging healthier choices in a hospital cafeteria: Results from a field study. *Health promotion practice, 19*(6), 925-934.

Meyer, K. A., Guilkey, D. K., Ng, S. W., Duffey, K. J., Popkin, B. M., Kiefe, C. I., . . . Gordon-Larsen, P. (2014). Sociodemographic differences in fast food price sensitivity. *JAMA internal medicine, 174*(3), 434-442.

Meyers, A. W., & Stunkard, A. J. (1980). Food accessibility and food choice: A test of Schachter's externality hypothesis. *Archives of General Psychiatry, 37*(10), 1133-1135.

Mhurchu, C. N., Blakely, T., Funaki-Tahifote, M., McKerchar, C., Wilton, J., Chua, S., & Jiang, Y. (2009). Inclusion of indigenous and ethnic minority populations in intervention trials: challenges and strategies in a New Zealand supermarket study. *Journal of Epidemiology and Community Health, 63*(10), 850-855.

Mhurchu, C. N., Blakely, T., Wall, J., Rodgers, A., Jiang, Y., & Wilton, J. (2007). Strategies to promote healthier food purchases: a pilot supermarket intervention study. *Public health nutrition, 10*(06), 608-615.

Michels, K. B., Bloom, B. R., Riccardi, P., Rosner, B. A., & Willett, W. C. (2008). A study of the importance of education and cost incentives on individual food choices at the Harvard School of Public Health cafeteria. *Journal of the American College of Nutrition, 27*(1), 6-11.

Miller, E. G., & Kahn, B. E. (2005). Shades of meaning: the effect of color and flavor names on consumer choice. *Journal of Consumer Research, 32*(1), 86-92.

Miller, G. F., Gupta, S., Kropp, J. D., Grogan, K. A., & Mathews, A. (2016). The effects of pre-ordering and behavioral nudges on National School Lunch Program participants' food item selection. *Journal of Economic Psychology, 55*, 4-16.

Mishra, A., Mishra, H., & Masters, T. M. (2012). The influence of bite size on quantity of food consumed: a field study. *Journal of consumer research, 38*(5), 791-795.

Mollen, S., Rimal, R. N., Ruiter, R. A. C., & Kok, G. (2013). Healthy and unhealthy social norms and food selection. Findings from a field-experiment. *Appetite, 65*, 83-89.

Morales, A., Kahn, B. E., McAlister, L., & Broniarczyk, S. M. (2005). Perceptions of assortment variety: The effects of congruency between consumers' internal and retailers' external organization. *Journal of Retailing, 81*(2), 159-169.

Moreau, P., Krishna, A., & Harlam, B. (2002). The manufacturer-retailer-consumer triad: Differing perceptions regarding price promotions. *Journal of Retailing, 77*(4), 547-569.

Morizet, D., Depezay, L., Combris, P., Picard, D., & Giboreau, A. (2012). Effect of labeling on new vegetable dish acceptance in preadolescent children. *Appetite, 59*(2), 399-402.

Morton, F. S., & Zettelmeyer, F. (2004). The strategic positioning of store brands in retailer–manufacturer negotiations. *Review of Industrial Organization, 24*(2), 161-194.

Muller, T. E. (1984). Buyer response to variations in product information load. *Journal of applied psychology, 69*(2), 300.

Muller, T. E. (1984). The use of nutritive composition data at the point of purchase. *Journal of nutrition education, 16*(3), 137-141.

Mullis, R., & Pirie, P. (1988). Lean meats make the grade--a collaborative nutrition intervention program. *Journal of the American Dietetic Association, 88*(2), 191-195.

Mullis, R. M., Hunt, M. K., Foster, M., Hachfeld, L., Lansing, D., Snyder, P., & Pirie, P. (1987). The shop smart for your heart grocery program. *Journal of Nutrition Education, 19*(5), 225-228.

Nakamura, R., Pechey, R., Suhrcke, M., Jebb, S. A., & Marteau, T. M. (2014). Sales impact of displaying alcoholic and non-alcoholic beverages in end-of-aisle locations: An observational study. *Social science & medicine, 108*, 68-73.

Nash, B., Booth-Butterfield, S., & Cooper, L. (2005). The cost-effectiveness of 1% or less media campaigns promoting low-fat milk consumption.

Nederkoorn, C., Havermans, R. C., Giesen, J. C., & Jansen, A. (2011). High tax on high energy dense foods and its effects on the purchase of calories in a supermarket. An experiment. *Appetite, 56*(3), 760-765. doi:https://doi.org/10.1016/j.appet.2011.03.002

Nguyen, M. T. T., Emberger-Klein, A., & Menrad, K. (2019). A Systematic Review on the Effects of Personalized Price Promotions for Food Products. *Journal of Food Products Marketing, 25*(3), 257-275. doi:10.1080/10454446.2018.1529647

Ni Mhurchu, C., Volkova, E., Jiang, Y., Eyles, H., Michie, J., Neal, B., . . . Rayner, M. (2017). Effects of interpretive nutrition labels on consumer food purchases: the Starlight randomized controlled trial. *The American journal of clinical nutrition, 105*(3), 695-704.

Nikolova, H. D., & Inman, J. J. (2015). Healthy Choice: The Effect of Simplified Point-of-Sale Nutritional Information on Consumer Food Choice Behavior. *Journal of Marketing Research, 52*(6), 817-835. doi:http://www.marketingpower.com/jmr

Novotny, R., Vijayadeva, V., Ramirez, V., Lee, S. K., Davison, N., & Gittelsohn, J. (2011). Development and Implementation of a Food System Intervention to Prevent Childhood Obesity in Rural Hawai 'i. *Hawaii medical journal, 70*(7 suppl 1), 42.

Närhinen, M., Nissinen, A., & Puska, P. (2000). Changes in supermarket sales during and after a staged health promotion campaign. *British Food Journal, 102*(4), 308-319. doi:10.1108/00070700010327733

O'Loughlin, J., Ledoux, J., Barnett, T., & Paradis, G. (1996). La Commande du Coeur ("Shop for Your Heart"): a point-of-choice nutrition education campaign in a low-income urban neighborhood. *American Journal of Health Promotion, 10*(3), 175-178.

O'Loughlin, J., Renaud, L., Richard, L., Gomez, L. S., & Paradis, G. (1998). Correlates of the sustainability of community-based heart health promotion interventions. *Preventive Medicine, 27*(5), 702-712.

Odenkirchen, J., Portnoy, B., Blair, J., Rodgers, A., Light, L., & Tenney, J. (1992). In-store monitoring of a supermarket nutrition intervention. *Fam Community Health, 14*(4), 1-9.

Ogawa, Y., Tanabe, N., Honda, A., Azuma, T., Seki, N., Suzuki, T., & Suzuki, H. (2011). Point-of-purchase health information encourages customers to purchase vegetables: Objective analysis by using a point-of-sales system. *Environmental Health and Preventive Medicine, 16*(4), 239-246. doi:10.1007/s12199-010-0192-8

Olson, C. M., Bisogni, C. A., & Thonney, P. F. (1982). Evaluation of a supermarket nutrition education program. *Journal of Nutrition Education, 14*(4), 141-145.

Olstad, D. L., Goonewardene, L. A., McCargar, L. J., & Raine, K. D. (2014). Choosing healthier foods in recreational sports settings: a mixed methods investigation of the impact of nudging and an economic incentive. *International Journal of Behavioral Nutrition and Physical Activity, 11*(1), 6.

Olstad, D. L., Vermeer, J., McCargar, L. J., Prowse, R. J. L., & Raine, K. D. (2015). Using traffic light labels to improve food selection in recreation and sport facility eating environments. *Appetite, 91*, 329-335.

Paine-Andrews, A., Francisco, V. T., Fawcett, S. B., Johnston, J., & Coen, S. (1997). Health marketing in the supermarket: Using prompting, product sampling, and price reduction to increase customer purchases of lower-fat items. *Health Marketing Quarterly, 14*(2), 85-99. doi:10.1300/J026v14n02_08

Paradis, G., O'Loughlin, J., Elliott, M., Masson, P., Renaud, L., Sacks-Silver, G., & Lampron, G. (1995). Coeur en santé St-Henri--a heart health promotion programme in a low income, low education neighbourhood in Montreal, Canada: theoretical model and early field experience. *Journal of Epidemiology and Community Health, 49*(5), 503-512.

Paton, C., Smith, F., Fraser, J., & McCormack, A. E. (1996). Shopping for health - food for thought: A programme in public health promotion. *Nutrition & Food Science, 96*(1), 11-13. doi:10.1108/00346659610105824

Pauwels, K., & Srinivasan, S. (2004). Who benefits from store brand entry? *Marketing Science, 23*(3), 364-390.

Payne, C. R., Niculescu, M., Just, D. R., & Kelly, M. P. (2015). Shopper marketing nutrition interventions: Social norms on grocery carts increase produce spending without increasing shopper budgets. *Preventive Medicine Reports, 2*, 287-291. doi:10.1016/j.pmedr.2015.04.007

Payne Riches, S., Aveyard, P., Piernas, C., Rayner, M., & Jebb, S. A. (2019). Optimising swaps to reduce the salt content of food purchases in a virtual online supermarket: a randomised controlled trial. *Appetite, 133*, 378-386. doi:10.1016/j.appet.2018.11.028

Perry, C. L., Bishop, D. B., Taylor, G. L., Davis, M., Story, M., Gray, C. W., . . . Harnack, L. (2004). A randomized school trial of environmental strategies to encourage fruit and vegetable consumption among children. *Health education & behavior, 31*(1), 65-76.

Phipps, E. J., Braitman, L. E., Stites, S. D., Singletary, S. B., Wallace, S. L., Hunt, L., . . . Uplinger, N. (2015). Impact of a Rewards-Based Incentive Program on Promoting Fruit and Vegetable Purchases. *American journal of public health, 105*(1), 166-172. Retrieved from http://ovidsp.ovid.com/ovidweb.cgi?T=JS&PAGE=reference&D=medp&NEWS=N&AN=24625144

Phipps, E. J., Kumanyika, S. K., Stites, S. D., Singletary, S. B., Cooblall, C., & DiSantis, K. I. (2014). Peer Reviewed: Buying Food on Sale: A Mixed Methods Study With Shoppers at an Urban Supermarket, Philadelphia, Pennsylvania, 2010–2012. *Preventing chronic disease, 11*.

Policastro, P., Palm, T., Schwartz, J., & Chapman, G. E. (2017). Targeted calorie message promotes healthy beverage consumption better than charity incentive. *Obesity, 25*(8), 1428-1434.

Policastro, P., Smith, Z., & Chapman, G. E. (2017). Put the healthy item first: Order of ingredient listing influences consumer selection. *Journal of health psychology, 22*(7), 853-863.

Powell, L. M., & Bao, Y. (2009). Food prices, access to food outlets and child weight. *Economics & Human Biology, 7*(1), 64-72.

Powell, L. M., & Han, E. (2011). Adult obesity and the price and availability of food in the United States. *American Journal of Agricultural Economics, 93*(2), 378-384.

Probart, C. K. (1993). In-store consumer nutrition education utilizing student educators. *Journal of Nutrition Education, 25*(1), 25-28.

Pulos, E., & Leng, K. (2010). Evaluation of a voluntary menu-labeling program in full-service restaurants. *American journal of public health, 100*(6), 1035-1039.

Raju, J. S., Sethuraman, R., & Dhar, S. K. (1995). The introduction and performance of store brands. *Management Science, 41*(6), 957-978.

Redden, J. P., Mann, T., Vickers, Z., Mykerezi, E., Reicks, M., & Elsbernd, S. (2015). Serving first in isolation increases vegetable intake among elementary schoolchildren. *PLoS One, 10*(4), e0121283.

Reger, B., Wootan, M. G., & Booth-Butterfield, S. (2000). A comparison of different approaches to promote community-wide dietary change. *American journal of preventive medicine, 18*(4), 271-275.

Reger, B., Wootan, M. G., Booth-Butterfield, S., & Smith, H. (1998). 1% or less: a community-based nutrition campaign. *Public health reports, 113*(5), 410.

Reicks, M., Redden, J. P., Mann, T., Mykerezi, E., & Vickers, Z. (2012). Photographs in lunch tray compartments and vegetable consumption among children in elementary school cafeterias. *Jama, 307*(8), 784-785.

Reicks, M., Splett, P., & Fishman, A. (1997). *Shelf labeling of organic foods: effects on customer perceptions and sales*: Retail Food Industry Center, University of Minnesota.

Reid, R. D., D'Angelo, M. E. S., Dombrow, C. A., Heshka, J. T., & Dean, T. R. (2004). The heart and stroke foundation of canada's health check food information program: Modelling program effects on consumer behaviour and dietary practices. *Canadian Journal of Public Health/Revue Canadienne de Sante'e Publique*, 146-150.

Rettie, R., & Brewer, C. (2000). The verbal and visual components of package design. *Journal of product & brand management, 9*(1), 56-70.

Roberto, C. A., Larsen, P. D., Agnew, H., Baik, J., & Brownell, K. D. (2010). Evaluating the impact of menu labeling on food choices and intake. *American journal of public health, 100*(2), 312-318.

Rodgers, A. B., Kessler, L. G., Portnoy, B., Potosky, A. L., Patterson, B., Tenney, J., . . . Mathews, O. (1994). " Eat for Health": a supermarket intervention for nutrition and cancer risk reduction. *American journal of public health, 84*(1), 72-76.

Rose, D., Hutchinson, P. L., Bodor, J. N., Swalm, C. M., Farley, T. A., Cohen, D. A., & Rice, J. C. (2009). Neighborhood food environments and body mass index: the importance of in-store contents. *American journal of preventive medicine, 37*(3), 214-219.

Rosecrans, A., Gittelsohn, J., Ho, L., Harris, S., Naqshbandi, M., & Sharma, S. (2008). Process evaluation of a multi-institutional community-based program for diabetes prevention among First Nations. *Health education research, 23*(2), 272-286.

Rozin, P., Scott, S. E., Dingley, M., Urbanek, J. K., Jiang, H., & Kaltenbach, M. (2011). Nudge to nobesity I: Minor changes in accessibility decrease food intake. *Judgment and Decision making, 6*(4), 323-332. Retrieved from https://repository.upenn.edu/marketing_papers/410

Russo, J. E., Staelin, R., Nolan, C. A., Russell, G. J., & Metcalf, B. L. (1986). Nutrition information in the supermarket. *Journal of Consumer Research, 13*(1), 48-70.

Sacks, G., Tikellis, K., Millar, L., & Swinburn, B. (2011). Impact of 'traffic-light'nutrition information on online food purchases in Australia. *Australian and New Zealand Journal of Public Health, 35*(2), 122-126.

Samuels, S. E., Craypo, L., Boyle, M., Crawford, P. B., Yancey, A., & Flores, G. (2010). The California endowment's healthy eating, active communities program: a midpoint review. *American journal of public health, 100*(11), 2114-2123.

Sanchez-Flack, J., Pickrel, J. L., Belch, G., Lin, S. F., Anderson, C. A. M., Martinez, M. E., . . . Ayala, G. X. (2017). Examination of the Relationship between In-Store Environmental Factors and Fruit and Vegetable Purchasing among Hispanics. *International journal of environmental research and public health, 14*(11). doi:10.3390/ijerph14111305

Schucker, R. E., Levy, A. S., Tenney, J. E., & Mathews, O. (1992). Nutrition shelf-labeling and consumer purchase behavior. *Journal of nutrition education, 24*(2), 75-81.

Schultz, J., & Litchfield, R. (2016). Evaluation of Traditional and Technology-Based Grocery Store Nutrition Education. *American Journal of Health Education, 47*(6), 355-364. doi:10.1080/19325037.2016.1219286

Schwartz, J., Riis, J., Elbel, B., & Ariely, D. (2012). Inviting consumers to downsize fast-food portions significantly reduces calorie consumption. *Health Affairs, 31*(2), 399-407.

Schwartz, M. B. (2007). The influence of a verbal prompt on school lunch fruit consumption: a pilot study. *International Journal of Behavioral Nutrition and Physical Activity, 4*(1), 6.

Scott, J. A., Begley, A. M., Miller, M. R., & Binns, C. W. (1991). Nutrition education in supermarkets: the Lifestyle 2000 experience. *Australian journal of public health, 15*(1), 49-55.

Scott, V., & Worsley, A. F. (1994). Ticks, claims, tables and food groups: a comparison for nutrition labelling. *Health Promotion International, 9*(1), 27-37.

Shah, A. M., Bettman, J. R., Ubel, P. A., Keller, P. A., & Edell, J. A. (2014). Surcharges plus unhealthy labels reduce demand for unhealthy menu items. *Journal of Marketing Research, 51*(6), 773-789.

Shannon, B., Mullis, R. M., Pirie, P. L., & Pheley, A. M. (1990). Promoting better nutrition in the grocery store using a game format: the shop smart game project. *Journal of Nutrition Education, 22*(4), 183-188.

Sharma, S., Sharma, S., Cao, X., Sharma, S., Cao, X., Arcan, C., . . . Mattingly, M. (2009). Assessment of dietary intake in an inner-city African American population and development of a quantitative food frequency questionnaire to highlight foods and nutrients for a nutritional invention. *International Journal of Food Sciences and Nutrition, 60*(sup5), 155-167.

Sigurdsson, V., Larsen, N. M., & Gunnarsson, D. (2014). Healthy food products at the point of purchase: An in-store experimental analysis. *Journal of applied behavior analysis, 47*(1), 151-154.

Silayoi, P., & Speece, M. (2007). The importance of packaging attributes: a conjoint analysis approach. *European Journal of Marketing, 41*(11/12), 1495-1517.

Sirohi, N., McLaughlin, E. W., & Wittink, D. R. (1998). A model of consumer perceptions and store loyalty intentions for a supermarket retailer. *Journal of Retailing, 74*(2), 223-245.

Smith, C., Parnell, W. R., Brown, R. C., & Gray, A. R. (2013). Providing additional money to food-insecure households and its effect on food expenditure: a randomized controlled trial. *Public health nutrition, 16*(8), 1507-1515.

Smith-Drelich, N. (2016). Buying health: assessing the impact of a consumer-side vegetable subsidy on purchasing, consumption and waste. *Public health nutrition, 19*(3), 520-529. doi:10.1017/S1368980015001469

Song, H.-J., Gittelsohn, J., Kim, M., Suratkar, S., Sharma, S., & Anliker, J. (2009). A corner store intervention in a low-income urban community is associated with increased availability and sales of some healthy foods. *Public health nutrition, 12*(11), 2060-2067.

Song, H.-J., Gittelsohn, J., Kim, M., Suratkar, S., Sharma, S., & Anliker, J. (2011). Korean American storeowners' perceived barriers and motivators for implementing a corner store-based program. *Health Promotion Practice, 12*(3), 472-482.

Soriano, E., & Dozier, D. M. (1978). Selling nutrition and heart-healthy diet behavior at the point-of-purchase. *Journal of Applied Nutrition*.

Steenhuis, I., Kroeze, W., Vyth, E., Valk, S., Verbauwen, R., & Seidell, J. (2010). The effects of using a nutrition logo on consumption and product evaluation of a sweet pastry. *Appetite, 55*(3), 707-709.

Steenhuis, I., Van Assema, P., Reubsaet, A., & Kok, G. (2004). Process evaluation of two environmental nutrition programmes and an educational nutrition programme conducted at supermarkets and worksite cafeterias in the Netherlands. *Journal of Human Nutrition and Dietetics, 17*(2), 107-115.

Steenhuis, I., van Assema, P., van Breukelen, G., & Glanz, K. (2004). The effectiveness of nutrition education and labeling in Dutch supermarkets. *American Journal of Health Promotion, 18*(3), 221-224.

Steenhuis, I., van Assema, P., van Breukelen, G., Glanz, K., Kok, G., & de Vries, H. (2004). The impact of educational and environmental interventions in Dutch worksite cafeterias. *Health Promotion International, 19*(3), 335-343.

Surkan, P. J., Tabrizi, M. J., Lee, R. M., Palmer, A. M., & Frick, K. D. (2016). Eat Right–Live well! supermarket intervention impact on sales of healthy foods in a low-income neighborhood. *Journal of nutrition education and behavior, 48*(2), 112-121. e111.

Sutherland, L. A., Kaley, L. A., & Fischer, L. (2010). Guiding stars: the effect of a nutrition navigation program on consumer purchases at the supermarket. *The American journal of clinical nutrition, 91*(4), 1090S-1094S.

Synovate, o. b. o. t. C. O. o. I., Food Standards Agency. (2005). *Quantitative Evaluation of Alternative Food Signposting Concepts: Report of Findings.* Retrieved from West Malling, UK https://webarchive.nationalarchives.gov.uk/20120404005151/http://www.food.gov.uk/multimedia/pdfs/signpostquanresearch.pdf

Saarela, A. M. (2014). Change of behaviour when selecting food products in a supermarket environment after reminding consumers about weight management. *Public health nutrition, 17*(5), 1147-1155. doi:10.1017/S136898001300044X

Tal, A., & Wansink, B. (2015). An Apple a Day Brings More Apples Your Way: Healthy Samples Prime Healthier Choices. *Psychology & Marketing, 32*(5), 575-584. doi:10.1002/mar.20801

Tandon, P. S., Zhou, C., Chan, N. L., Lozano, P., Couch, S. C., Glanz, K., . . . Saelens, B. E. (2011). The impact of menu labeling on fast-food purchases for children and parents. *American journal of preventive medicine, 41*(4), 434-438.

Teisl, M. F., & Levy, A. S. (1997). Does nutrition labeling lead to healthier eating? *Journal of Food Distribution Research, 28*, 18-27.

Thapa, J., Lyford, C., Belasco, E. J., McCool, B., McCool, A., Pence, B., & Carter, T. (2014). *Nudges in the Supermarket: Experience from Point of Sale Signs. 2014 Annual Meeting, July 27-29, 2014*. Paper presented at the Agricultural and Applied Economics Association, Minneapolis, Minnesota 171442.

The National Heart Foundation of Australia. (2009). Australians and Front of Pack Labelling: What We Want, What We Need. Retrieved from www.heartfoundation.org.au/SiteCollectionDocuments/Tick-Heart-Foundation-Research-Summary-FOPL.pdf

Thomas, J. M., Ursell, A., Robinson, E. L., Aveyard, P., Jebb, S. A., Herman, C. P., & Higgs, S. (2017). Using a descriptive social norm to increase vegetable selection in workplace restaurant settings. *Health psychology, 36*(11), 1026.

Thorndike, A., Levy, D. E., Macias-Navarro, L., Franckle, R. L., & Rimm, E. B. (2015). Traffic-light labels and financial incentives reduce purchase of sugar-sweetened beverages by low-income, Latino families: a randomized controlled trial. *Circulation, 131*. Retrieved from https://www.cochranelibrary.com/central/doi/10.1002/central/CN-01753558/full

Thorndike, A. N., Riis, J., Sonnenberg, L. M., & Levy, D. E. (2014). Traffic-light labels and choice architecture: promoting healthy food choices. *American journal of preventive medicine, 46*(2), 143-149.

Thorndike, A. N., Sonnenberg, L., Riis, J., Barraclough, S., & Levy, D. E. (2012). A 2-phase labeling and choice architecture intervention to improve healthy food and beverage choices. *American journal of public health, 102*(3), 527-533.

Thow, A. M., Downs, S., & Jan, S. (2014). A systematic review of the effectiveness of food taxes and subsidies to improve diets: understanding the recent evidence. *Nutr. Rev., 72*(9), 551-565. doi:https://doi.org/10.1111/nure.12123

Turnwald, B. P., Boles, D. Z., & Crum, A. J. (2017). Association between indulgent descriptions and vegetable consumption: Twisted carrots and dynamite beets. *JAMA internal medicine, 177*(8), 1216-1218.

Vadiveloo, M. K., Guan, X., Parker, H. W., Perraud, E., Buchanan, A., Atlas, S., & Thorndike, A. N. (2020). Evaluating the effect of individually-targeted food incentives on grocery purchases: The smart cart study protocol for a randomized controlled cross-over trial. *Contemporary clinical trials, 91*, 105966. doi:https://dx.doi.org/10.1016/j.cct.2020.105966

van Assema, P., Brug, J., Glanz, K., Dolders, M., & Mudde, A. (1998). Nationwide implementation of guided supermarket tours in the Netherlands: a dissemination study. *Health education research, 13*(4), 557-566.

Van Assema, P., Steenbakkers, M., Stapel, H., Van Keulen, H., Ronda, G., & Brug, J. (2006). Evaluation of a Dutch public-private partnership to promote healthier diet. *American Journal of Health Promotion, 20*(5), 309-312.

Van Ittersum, K., & Wansink, B. (2013). Extraverted children are more biased by bowl sizes than introverts. *PLoS One, 8*(10), e78224.

van Kleef, E., van den Broek, O., & van Trijp, H. C. M. (2015). Exploiting the spur of the moment to enhance healthy consumption: Verbal prompting to increase fruit choices in a self-service restaurant. *Applied Psychology: Health and Well-Being, 7*(2), 149-166.

Van Kleef, E., Van Trijp, H., Paeps, F., & Fernández-Celemín, L. (2008). Consumer preferences for front-of-pack calories labelling. *Public health nutrition, 11*(02), 203-213.

Vandenbroele, J., Slabbinck, H., Van Kerckhove, A., & Vermeir, I. (2018). Curbing portion size effects by adding smaller portions at the point of purchase. *Food Quality and Preference, 64*, 82-87. doi:10.1016/j.foodqual.2017.10.015

Vanderlee, L., & Hammond, D. (2014). Does nutrition information on menus impact food choice? Comparisons across two hospital cafeterias. *Public health nutrition, 17*(6), 1393-1402.

Vasiljevic, M., Cartwright, E., Pilling, M., Lee, M.-M., Bignardi, G., Pechey, R., . . . Marteau, T. M. (2018). Impact of calorie labelling in worksite cafeterias: a stepped wedge randomised controlled pilot trial. *International journal of behavioral nutrition and physical activity, 15*(1), 41.

Volkova, E., Neal, B., Rayner, M., Swinburn, B., Eyles, H., Jiang, Y., . . . Ni Mhurchu, C. (2014). Effects of interpretive front-of-pack nutrition labels on food purchases: protocol for the Starlight randomised controlled trial. *BMC public health, 14*, 968. doi:10.1186/1471-2458-14-968

von Philipsborn, P., Stratil, J. M., Burns, J., Busert, L. K., Pfadenhauer, L. M., Polus, S., . . . Rehfuess, E. (2019). Environmental interventions to reduce the consumption of sugar-sweetened beverages and their effects on health. *Cochrane Database of Systematic Reviews*(6). doi:10.1002/14651858.CD012292.pub2

Vyth, E. L., Steenhuis, I. H., Mallant, S. F., Mol, Z. L., Brug, J., Temminghoff, M., . . . Seidell, J. C. (2009). A front-of-pack nutrition logo: a quantitative and qualitative process evaluation in the Netherlands. *Journal of health communication, 14*(7), 631-645.

Vyth, E. L., Steenhuis, I. H., Vlot, J. A., Wulp, A., Hogenes, M. G., Looije, D. H., . . . Seidell, J. C. (2010). Actual use of a front-of-pack nutrition logo in the supermarket: consumers' motives in food choice. *Public health nutrition, 13*(11), 1882-1889.

Wagner, J. L., Winett, R. A., & Walbert-Rankin, J. (1992). Influences of a supermarket intervention on the food choices of parents and their children. *Journal of nutrition education, 24*(6), 306-311.

Walters, R., & Jamil, M. (2002). Measuring cross-category specials purchasing: theory, empirical results, and implications. *Journal of Market-Focused Management, 5*(1), 25-42.

Wanke, M., Lehmann, G., & Bless, H. (1997). *The In and Out of Canned Soup: Brand Management by Influencing the Categorization of Brand Exemplars—An Inclusion/Exclusion Approach.* Paper presented at the Society for Consumer Psychology.

Wansink, B. (1996). Can package size accelerate usage volume? *The Journal of Marketing*, 1-14.

Wansink, B. (2004). Environmental factors that increase the food intake and consumption volume of unknowing consumers. *Annu. Rev. Nutr., 24*, 455-479.

Wansink, B., Bhana, H., Qureshi, M., & Cadenhead, J. W. (2016). Using choice architecture to create healthy food interventions in food pantries. *Journal of nutrition education and behavior, 48*(7), S35-S36.

Wansink, B., & Chandon, P. (2006). Can "low-fat" nutrition labels lead to obesity? *Journal of Marketing Research, 43*(4), 605-617.

Wansink, B., & Chandon, P. (2014). Slim by design: Redirecting the accidental drivers of mindless overeating. *Journal of Consumer Psychology, 24*(3), 413-431.

Wansink, B., & Hanks, A. S. (2013). Slim by design: serving healthy foods first in buffet lines improves overall meal selection. *PLoS ONE, 8*(10), e77055.

Wansink, B., Kent, R. J., & Hoch, S. J. (1998). An anchoring and adjustment model of purchase quantity decisions. *Journal of Marketing Research*, 71-81.

Wansink, B., & Kim, J. (2005). Bad popcorn in big buckets: portion size can influence intake as much as taste. *Journal of Nutrition Education and Behavior, 37*(5), 242-245.

Wansink, B., & Payne, C. R. (2007). Counting bones: environmental cues that decrease food intake. *Perceptual and Motor Skills, 104*(1), 273-276.

Wansink, B., Soman, D., & Herbst, K. C. (2017). Larger partitions lead to larger sales: Divided grocery carts alter purchase norms and increase sales. *Journal of Business Research, 75*, 202-209.

Wansink, B., & Van Ittersum, K. (2013). Portion size me: Plate-size induced consumption norms and win-win solutions for reducing food intake and waste. *Journal of Experimental Psychology: Applied, 19*(4), 320.

Wansink, B., Van Ittersum, K., & Painter, J. E. (2006). Ice cream illusions: bowls, spoons, and self-served portion sizes. *American journal of preventive medicine, 31*(3), 240-243.

Wansink, B., Van Ittersum, K., & Payne, C. R. (2014). Larger bowl size increases the amount of cereal children request, consume, and waste. *The Journal of pediatrics, 164*(2), 323-326.

Waterlander, W. E., Mhurchu, C. N., & Steenhuis, I. H. (2014). Effects of a price increase on purchases of sugar sweetened beverages. Results from a randomized controlled trial. *Appetite, 78*, 32-39.

Waterlander, W. E., Steenhuis, I. H., de Boer, M. R., Schuit, A. J., & Seidell, J. C. (2012). Introducing taxes, subsidies or both: the effects of various food pricing strategies in a web-based supermarket randomized trial. *Preventive medicine, 54*(5), 323-330. doi:10.1016/j.ypmed.2012.02.009

Waterlander, W. E., Steenhuis, I. H., de Boer, M. R., Schuit, A. J., & Seidell, J. C. (2013). Effects of different discount levels on healthy products coupled with a healthy choice label, special offer label or both: results from a web-based supermarket experiment. *International Journal of Behavioral Nutrition and Physical Activity, 10*(1), 59.

Waterlander, W. E., Steenhuis, I. H., de Vet, E., Schuit, A. J., & Seidell, J. C. (2010). Expert views on most suitable monetary incentives on food to stimulate healthy eating. *The European Journal of Public Health, 20*(3), 325-331.

Waterlander, W. E., Steenhuis, I. H. M., de Boer, M. R., Schuit, A. J., & Seidell, J. C. (2012). The effects of a 25% discount on fruits and vegetables: results of a randomized trial in a three-dimensional web-based supermarket. *International journal of behavioral nutrition and physical activity, 9*(1), 11.

Webb, K. L., Solomon, L. S., Sanders, J., Akiyama, C., & Crawford, P. B. (2011). Menu labeling responsive to consumer concerns and shows promise for changing patron purchases. *Journal of Hunger & Environmental Nutrition, 6*(2), 166-178.

Wendt, M., & Todd, J. (2011). The Effect of Food and Beverage Prices on Children's Weights. Washington: US Department of Agriculture. *Economic Research Service*.

Which? Consumers' Association. (2006). Healthy Signs? Campaign Report. Retrieved from http://www.which.co.uk/documents/pdf/healthy-signs-whichreport-176909.pdf.

Wilson, N. L. W., Just, D. R., Swigert, J., & Wansink, B. (2017). Food pantry selection solutions: a randomized controlled trial in client-choice food pantries to nudge clients to targeted foods. *Journal of Public Health, 39*(2), 366-372.

Winett, R. A., Kramer, K. D., Walker, W. B., Malone, S. W., & Lane, M. (1988). Modifying food purchases in supermarkets with modeling, feedback, and goal-setting procedures. *Journal of Applied Behavior Analysis, 21*(1), 73-80.

Winett, R. A., Moore, J. F., Wagner, J. L., Hite, L. A., Leahy, M., Neubauer, T. E., . . . Geller, E. S. (1991). Altering Shoppers´s Supermarket Purchases to fit Nutrition Guidelines: An interactive Information System. *Journal of Applied Behavior Analysis, 24*(1), 95-105.

Winkler, L. L., Christensen, U., Glumer, C., Bloch, P., Mikkelsen, B. E., Wansink, B., & Toft, U. (2016). Substituting sugar confectionery with fruit and healthy snacks at checkout - a win-win strategy for consumers and food stores? a study

on consumer attitudes and sales effects of a healthy supermarket intervention. *BMC public health, 16*. doi:10.1186/s12889-016-3849-4

Yang, C.-C., & Chiou, W.-B. (2010). Substitution of healthy for unhealthy beverages among college students. A health-concerns and behavioral-economics perspective. *Appetite, 54*(3), 512-516.

Yao, J., Oppewal, H., & Wang, D. (2020). Cheaper and smaller or more expensive and larger: how consumers respond to unit price increase tactics that simultaneously change product price and package size. *Journal of the Academy of Marketing Science*. doi:10.1007/s11747-019-00716-z

Zhang, Z. J., Krishna, A., & Dhar, S. K. (2000). The optimal choice of promotional vehicles: Front-loaded or rear-loaded incentives? *Management Science, 46*(3), 348-362.

Zielke, S., & Toporowski, W. (2009). Does architecture influence the price image and intention to shop in a retail store? In *European retail research* (pp. 83-100): Springer.

Table S5. Risk of bias of included studies with support judgment

**Achabal et al., 1987**		
Study design	Randomized controlled trails	
**Bias**	**Autors´ judgment**	**Support for judgement**
Random sequence generation (selection bias)	Unclear risk	Insufficient detail about the sequence generation process. Unclear why some participants were control and others intervention.
Allocation concealment (selection bias)	Unclear risk	Not described in sufficient detail.
Blinding of participants and personnel (performance bias)	Low risk	Not clear if blinding but outcome not likely to be influenced by lack of blinding.
Blinding of outcome assessment (detection bias)	Low risk	Outcome was measured by raw sales data. Blinding of outcome assessment ensured.
Incomplete outcome data (attrition bias)	Unclear risk	Insufficient reporting on if any sites dropped out of if certain sales data were excluded.
Selective reporting (reporting bias)	Unclear risk	No description of why analysis was done for only selected products.
Other bias	Low risk	Study appears to be free of other sources of bias.
**Anderson et al., 1997**		
Study design	Randomized controlled trails	
**Bias**	**Autors´ judgment**	**Support for judgement**
Random sequence generation (selection bias)	Unclear risk	Insufficient detail about the sequence generation process. Unclear why some participants were control and others intervention group.
Allocation concealment (selection bias)	Unclear risk	Not described in sufficient detail.
Blinding of participants and personnel (performance bias)	High risk	Participants are approached by researcher in store asking if they want to be part of. Quote: "Research project with a program to help shoppers make healthier food purchases". Outcome is likely to be influenced by lack of blinding
Blinding of outcome assessment (detection bias)	Low risk	Quote: " Data-entry clerks, blind to treatment condition, used a data-entry application based on dBase IV software (Ashton-Tate, Corp., 1988) to enter data from supermarket receipts and participant enrollment forms." Comment: Outcome was measured by supermarket receipts. Blinding of outcome assessment ensured.
Incomplete outcome data (attrition bias)	Low risk	Missing outcome data balanced in number across intervention groups, with similar seasons for missing data across groups.
Selective reporting (reporting bias)	Unclear risk	Insufficient information to assess whether an important risk of bias exists. Study protocol is not available.

Other bias	Low risk	Study appears to be free of other sources of bias.
**Anderson et al., 2001**		
Study design	Randomized controlled trails	
**Bias**	**Autors´ judgment**	**Support for judgement**
Random sequence generation (selection bias)	Unclear risk	Insufficient detail about the sequence generation process. Unclear why some participants were control and others intervention group.
Allocation concealment (selection bias)	Unclear risk	Not described in sufficient detail.
Blinding of participants and personnel (performance bias)	Unclear risk	Not described in sufficient detail.
Blinding of outcome assessment (detection bias)	Low risk	Comment: Outcome was measured by supermarket receipts. Blinding of outcome assessment ensured.
Incomplete outcome data (attrition bias)	High risk	Imbalance in numbers for missing data across intervention groups. Only dropout in intervention group therefore likely to be related to intervention.
Selective reporting (reporting bias)	Unclear risk	Insufficient information to assess whether an important risk of bias exists. Study protocol is not available.
Other bias	Low risk	Study appears to be free of other sources of bias.
**Ayala et al., 2013**		
Study design	Randomized controlled trails	
**Bias**	**Autors´ judgment**	**Support for judgement**
Random sequence generation (selection bias)	Unclear risk	Insufficient detail about the sequence generation process. Unclear why some sites were control sites and others intervention.
Allocation concealment (selection bias)	Unclear risk	Not described in sufficient detail.
Blinding of participants and personnel (performance bias)	High risk	Researcher describes objective of study participants in both intervention and control group. Personnel not blinded but unlikely to impact outcome.
Blinding of outcome assessment (detection bias)	High risk	Outcome assessment not blind as participants were asked about effect of intervention via telephone interview.
Incomplete outcome data (attrition bias)	Low risk	Missing outcome data balanced in number across intervention groups, with similar seasons for missing data across groups.
Selective reporting (reporting bias)	Unclear risk	Insufficient information to assess whether an important risk of bias exists. Study protocol is not available.
Other bias	Low risk	Cluster randomization of sites was done prior to group assignment and the same participants were followed over time.
**Ball et al., 2015**		
Study design	Randomized controlled trails	
**Bias**	**Autors´ judgment**	**Support for judgement**
Random sequence generation (selection bias)	Low risk	Quote: "642 women were randomly assigned to one of 4 conditions by using a computer-generated block-randomization sequence produced". Comment: Random sequence generation ensured.
Allocation concealment (selection bias)	Low risk	Quote: "Allocation concealment was enabled via the secure storage of the randomization sequence separately from the participant database, which was accessible only by the data manager and statistician."

Blinding of participants and personnel (performance bias)	Low risk	Participants cannot foresee assignment to intervention or control group. Personnel not blinded but unlikely to impact outcome.
Blinding of outcome assessment (detection bias)	Low risk	Outcome was measured by supermarket transaction data. Blinding of outcome assessment ensured.
Incomplete outcome data (attrition bias)	High risk	Imbalance in numbers for missing data across intervention groups. Only dropout in intervention group therefore likely to be related to intervention.
Selective reporting (reporting bias)	Low risk	Study protocol available and all of the study´s pre-specified outcomes that are of interest have been reported in the pre-specified way.
Other bias	High risk	Authors write that they want to do intent-to-treat analysis but do not report it in final study.
**Connell et al., 2001**		
Study design	Randomized controlled trails	
**Bias**	**Autors´ judgment**	**Support for judgement**
Random sequence generation (selection bias)	Unclear risk	Insufficient detail about the sequence generation process. Unclear why some participants were control and others intervention group.
Allocation concealment (selection bias)	Unclear risk	Not described in sufficient detail.
Blinding of participants and personnel (performance bias)	High risk	Intervention consisted of a task that participants had to at home. Not blinded and may impact results. Personnel not blinded and may impact outcome since outcome measurement was self-reported consumption.
Blinding of outcome assessment (detection bias)	High risk	Outcome assessment not blind as participants were asked about effect of intervention via telephone interview.
Incomplete outcome data (attrition bias)	Unclear risk	Insufficient reporting on if any participants dropped out.
Selective reporting (reporting bias)	Unclear risk	Insufficient information to assess whether an important risk of bias exists. Study protocol is not available.
Other bias	Unclear risk	Insufficient information to permit low or high risk
**Finkelstein et al., 2019**		
Study design	Randomized controlled trails	
**Bias**	**Autors´ judgment**	**Support for judgement**
Random sequence generation (selection bias)	Low risk	Quote: "Participants were randomly assigned to one of six intervention sequences via random permuted blocks of size three with equal allocation for the six sequences (...) by a computer program"
Allocation concealment (selection bias)	Unclear risk	Not described in sufficient detail.
Blinding of participants and personnel (performance bias)	Low risk	Participants cannot foresee assignment to intervention or control group. Personnel not blinded but unlikely to impact outcome.
Blinding of outcome assessment (detection bias)	Low risk	Outcome was measured by supermarket transaction data. Blinding of outcome assessment ensured.
Incomplete outcome data (attrition bias)	Low risk	Missing outcome data balanced in number across intervention groups, with similar seasons for missing data across groups.
Selective reporting (reporting bias)	Low risk	Study protocol available and all of the study´s pre-specified outcomes that are of interest have been reported in the pre-specified way.
Other bias	Low risk	Study appears to be free of other sources of bias.
**Finkelstein et al., 2020**		
Study design	Randomized controlled trails	

Bias	Autors´ judgment	Support for judgement
Random sequence generation (selection bias)	Unclear risk	Insufficient detail about the sequence generation process. Unclear why some participants were control and others intervention group.
Allocation concealment (selection bias)	Unclear risk	Not described in sufficient detail.
Blinding of participants and personnel (performance bias)	Unclear risk	Not described in sufficient detail.
Blinding of outcome assessment (detection bias)	Unclear risk	Not described in sufficient detail.
Incomplete outcome data (attrition bias)	Low risk	Missing outcome data balanced in number across intervention groups, with similar seasons for missing data across groups.
Selective reporting (reporting bias)	Unclear risk	Insufficient information to assess whether an important risk of bias exists. Study protocol is not available.
Other bias	Low risk	Study appears to be free of other sources of bias.
**Foster et al., 2014**		
Study design	Randomized controlled trails	
**Bias**	**Autors´ judgment**	**Support for judgement**
Random sequence generation (selection bias)	Unclear risk	Insufficient detail about the sequence generation process. Unclear why some sites were control sites and others intervention.
Allocation concealment (selection bias)	Low risk	Quote: "The randomization allocation sequence was created by a statistician and implemented by a research coordinator. Within each pair, the stores were randomly assigned to intervention or control."
Blinding of participants and personnel (performance bias)	Low risk	Not clear if blinding but outcome not likely to be influenced by lack of blinding. Personnel not blinded but unlikely to impact outcome.
Blinding of outcome assessment (detection bias)	Low risk	Outcome was measured by supermarket transaction data. Blinding of outcome assessment ensured.
Incomplete outcome data (attrition bias)	Low risk	No missing data as all transaction data was used in analysis.
Selective reporting (reporting bias)	Unclear risk	Insufficient information to assess whether an important risk of bias exists. Study protocol is not available.
Other bias	Low risk	Cluster randomization of sites was done prior to group assignment and the same participants were followed over time.
**Franckle et al., 2018**		
Study design	Randomized controlled trails	
**Bias**	**Autors´ judgment**	**Support for judgement**
Random sequence generation (selection bias)	Low risk	Quote: "The Stata statistical software package was used to randomize participants 1:1 to intervention or control in blocks of 8"
Allocation concealment (selection bias)	Unclear risk	Insufficient information.
Blinding of participants and personnel (performance bias)	Low risk	Quote: "Study participants and investigators were blinded to the randomization assignment during the baseline data collection months (...) and all participants were notified of their group assignment in April 2014." Comment: Participants cannot foresee assignment to intervention or control group. Personnel not blinded but unlikely to impact outcome.

Blinding of outcome assessment (detection bias)	Low risk	Outcome was measured by supermarket transaction data tracked trough store loyalty card. Blinding of outcome assessment ensured.
Incomplete outcome data (attrition bias)	Low risk	Missing outcome data balanced in number across intervention groups, with similar seasons for missing data across groups.
Selective reporting (reporting bias)	Unclear risk	Insufficient information to assess whether an important risk of bias exists. Study protocol is not available.
Other bias	Low risk	Study appears to be free of other sources of bias.

Geliebter et al., 2013

Study design	Randomized controlled trails	
**Bias**	**Autors´ judgment**	**Support for judgement**
Random sequence generation (selection bias)	Low risk	Quote: "Participants were first stratified by gender and then randomized within each store, using a randomizer program (random.org) into two groups"
Allocation concealment (selection bias)	Unclear risk	Insufficient information.
Blinding of participants and personnel (performance bias)	Low risk	Participants were blind to their group assignments during the 4- week baseline period, and were informed of their group assignment just before the 8-week intervention period began. Comment: Study was unblinded at the beginning of the study. Personell not blinded but unlikely to impact outcome but ulikely to influence outcome.
Blinding of outcome assessment (detection bias)	Low risk	Quote: "Participants were blind to their group assignments during the 4- week baseline period, and were informed of their group assignment just before the 8-week intervention period began." Comment: Participants know which group they were part of.
Incomplete outcome data (attrition bias)	High risk	Dropout rates did differ by study condition (discount: 7%: control: 30%), which could be due to participants dropping out when told just prior to the start of the intervention period that they were randomized into the control group, and therefore would not receive the discounts. Reason for missing outcome data likely to be related to true outcome, with either imbalance in numbers of reasons for missing data across intervention groups.
Selective reporting (reporting bias)	Unclear risk	Comment: Insufficient information to assess whether an important risk of bias exists. Study protocol is not available.
Other bias	Low risk	Study appears to be free of other sources of bias.

Huang et al., 2006

Study design	Randomized controlled trails	
**Bias**	**Autors´ judgment**	**Support for judgement**
Random sequence generation (selection bias)	Low risk	Quote: "Recruitment process was completed, participants were assigned to intervention or control at random by a central computerized process with minimization by age, sex, and number of individuals the food was being purchased for."
Allocation concealment (selection bias)	Unclear risk	Article does not say how concealment was done. Quote: "The participants were unaware of treatment allocation, and the principal investigator performed unblinding of the treatment allocation once the study follow-up had ended."
Blinding of participants and personnel (performance bias)	Low risk	Participants blinded until follow-up period. Personnel not blinded but unlikely to impact outcome.

Blinding of outcome assessment (detection bias)	Low risk	Based on the use of sales data.
Incomplete outcome data (attrition bias)	Low risk	Missing outcome data balanced in number across intervention groups, with similar seasons for missing data across groups.
Selective reporting (reporting bias)	Low risk	Study protocol available and all of the study´s pre-specified outcomes that are of interest have been reported in the pre-specified way.
Other bias	Low risk	Study appears to be free of other sources of bias.

Kristal et al., 1997

Study design	Randomized controlled trails	
**Bias**	**Autors´ judgment**	**Support for judgement**
Random sequence generation (selection bias)	Low risk	Quote: "Using tables of random numbers, participants were systematically selected" Comment: Randomization was by store and not individuals.
Allocation concealment (selection bias)	Unclear risk	Insufficient information.
Blinding of participants and personnel (performance bias)	Low risk	Participants cannot foresee assignment to intervention or control group. Personnel not blinded but unlikely to impact outcome.
Blinding of outcome assessment (detection bias)	High risk	Outcome assessment not blind as participants were asked about effect of intervention via take-home survey on intake of fruits and vegetables.
Incomplete outcome data (attrition bias)	Low risk	Missing outcome data balanced in number across intervention groups, with similar seasons for missing data across groups.
Selective reporting (reporting bias)	High risk	State in protocol that they wanted to use sales data as outcome measurement but was not possible therefore used self-reported data.
Other bias	Low risk	Cluster randomization of sites was done prior to group assignment and the same participants were followed over time.

Mhurchu et al., 2010

Study design	Randomized controlled trails	
**Bias**	**Autors´ judgment**	**Support for judgement**
Random sequence generation (selection bias)	Low risk	Quote: Strategies used were mail-outs to a random selection of customers who were registered to use the Shop 'N Go system." Comment: not likely that costumers registered are differently affected by intervention than costumers who are not registered.
Allocation concealment (selection bias)	Low risk	Quote: "Treatment assignment codes were not available to investigators or research staff at any point during the study."
Blinding of participants and personnel (performance bias)	Low risk	Quote: "Because participants were not blinded, it is possible that some of the effect of price discounts could have been due to displacement shopping from other locations into intervention supermarkets. However, our sensitivity analyses suggest the main study findings are robust."
Blinding of outcome assessment (detection bias)	Low risk	Based on the use of sales data.
Incomplete outcome data (attrition bias)	Low risk	Missing outcome data balanced in number across intervention groups, with similar seasons for missing data across groups.

Selective reporting (reporting bias)	Low risk	Study protocol available and all of the study´s pre-specified outcomes that are of interest have been reported in the pre-specified way.
Other bias	Low risk	Study appears to be free of other sources of bias.
**Milliron et al., 2012**		
Study design	Randomized controlled trails	
**Bias**	**Autors´ judgment**	**Support for judgement**
Random sequence generation (selection bias)	Low risk	Quote: "Participants were randomly assigned to either the intervention or control group using block randomization immediately after consenting to the study." Comment: Block randomized by time of the day and rotated then intervention took place
Allocation concealment (selection bias)	Low risk	Quote: "random number sequence generated with a computer program"
Blinding of participants and personnel (performance bias)	Low risk	Participants cannot foresee assignment to intervention or control group. Personnel not blinded but unlikely to impact outcome.
Blinding of outcome assessment (detection bias)	Low risk	Data was collected by digital photographs of all food and beverage purchases in shopping baskets
Incomplete outcome data (attrition bias)	High risk	Imbalance in numbers for missing data across intervention groups. Only dropout in intervention group therefore likely to be related to intervention. Dropout from treatment was 10 and control was 1, no information about why these were excluded from analysis.
Selective reporting (reporting bias)	Unclear risk	Insufficient information to assess whether an important risk of bias exists. Study protocol is not available.
Other bias	Low risk	Study appears to be free of other sources of bias.
**Papies et al., 2014**		
Study design	Randomized controlled trails	
**Bias**	**Autors´ judgment**	**Support for judgement**
Random sequence generation (selection bias)	Unclear risk	Insufficient detail about the sequence generation process. Unclear why some participants were control and others intervention.
Allocation concealment (selection bias)	Unclear risk	Not described in sufficient detail.
Blinding of participants and personnel (performance bias)	Low risk	Not clear if blinding but outcome not likely to be influenced by lack of blinding. Personnel not blinded but unlikely to impact outcome.
Blinding of outcome assessment (detection bias)	Low risk	Outcome was measured by supermarket receipts. Blinding of outcome assessment ensured.
Incomplete outcome data (attrition bias)	Low risk	Missing outcome data balanced in number across intervention groups, with similar seasons for missing data across groups.
Selective reporting (reporting bias)	High risk	Intervention was recipe flyer for healthy pasta, but outcome was measured on snacks. May have had selective reporting. Important limitation is that study only assessed snack purchases, rather than overall calories sales.
Other bias	Low risk	Study appears to be free of other sources of bias.
**Polacsek et al., 2018**		
Study design	Randomized controlled trails	
**Bias**	**Autors´ judgment**	**Support for judgement**
Random sequence generation (selection bias)	Low risk	Randomization at store level not individual. Gives explanation for why sites were treatment or control sites.

Allocation concealment (selection bias)	Unclear risk	Not described in sufficient detail.
Blinding of participants and personnel (performance bias)	Low risk	Not clear if blinding but outcome not likely to be influenced by lack of blinding. Personnel not blinded but unlikely to impact outcome.
Blinding of outcome assessment (detection bias)	Low risk	Participants were likely to be blinded as they did not know that they were part of experiment. Cashiers received information about the goals of the study but were not able to distinguish between control and intervention participants based on the loyalty card.
Incomplete outcome data (attrition bias)	Low risk	Missing outcome data balanced in number across intervention groups, with similar seasons for missing data across groups.
Selective reporting (reporting bias)	Unclear risk	Insufficient information to assess whether an important risk of bias exists. Study protocol is not available.
Other bias	Low risk	Study appears to be free of other sources of bias.

Salmon et al., 2015

Study design	Randomized controlled trails	
**Bias**	**Autors´ judgment**	**Support for judgement**
Random sequence generation (selection bias)	Unclear risk	Cluster randomized by time of the day but does not described in sufficient detail how randomization was ensured.
Allocation concealment (selection bias)	High risk	Researcher describes objective of study participants.
Blinding of participants and personnel (performance bias)	Low risk	Quote: "participants were invited to take part in an experiment that was ostensibly about the influence of the time of the day on concentration levels" Comment: Participants did not know that intervention was promotion of low-fat cheese. Personnel not blinded but unlikely to impact outcome.
Blinding of outcome assessment (detection bias)	Low risk	Quote: "After customers had passed the cashiers, we asked for their receipts, which specified the amount of money spent on the low-fat cheese." Comment: Blinding of outcome assessment ensured.
Incomplete outcome data (attrition bias)	Low risk	Missing outcome data balanced in number across intervention groups, with similar seasons for missing data across groups.
Selective reporting (reporting bias)	Unclear risk	Insufficient information to assess whether an important risk of bias exists. Study protocol is not available.
Other bias	Low risk	Cluster participants were recruited prior to group assignment and the same participants were followed over time.

Steenhuis et al., 2004

Study design	Randomized controlled trails	
**Bias**	**Autors´ judgment**	**Support for judgement**
Random sequence generation (selection bias)	Unclear risk	Insufficient detail about the sequence generation process. Unclear why some participants were control and others intervention.
Allocation concealment (selection bias)	Unclear risk	Not described in sufficient detail.
Blinding of participants and personnel (performance bias)	Low risk	Not clear if blinding but outcome not likely to be influenced by lack of blinding. Personnel not blinded but unlikely to impact outcome.

Blinding of outcome assessment (detection bias)	High risk	Quote: "Total fat intake was measured with a short food frequency list validated in earlier research" Comment: Self-reported intake as outcome measurement.
Incomplete outcome data (attrition bias)	Unclear risk	Numbers of respondents at posttest is unclear.
Selective reporting (reporting bias)	Unclear risk	Insufficient information to assess whether an important risk of bias exists. Study protocol is not available.
Other bias	Unclear risk	No info on conflict of interests.
**Thorndike et al., 2017**		
Study design	Randomized controlled trails	
**Bias**	**Autors´ judgment**	**Support for judgement**
Random sequence generation (selection bias)	Unclear risk	Insufficient detail about the sequence generation process. Unclear why some participants were control and others intervention.
Allocation concealment (selection bias)	Unclear risk	Not described in sufficient detail.
Blinding of participants and personnel (performance bias)	Low risk	Quote: «Stores were not told of their assignment until one month prior to implementation of the intervention in November 2013". Comment: We do not consider this impacting the results
Blinding of outcome assessment (detection bias)	Low risk	Stores were not told of their assignment until one month prior to implementation of the intervention in November 2013 but we do not consider this impacting the results.
Incomplete outcome data (attrition bias)	Unclear risk	Insufficient reporting on if any participants dropped out.
Selective reporting (reporting bias)	Unclear risk	Insufficient information to assess whether an important risk of bias exists. Study protocol is not available.
Other bias	Low risk	Study appears to be free of other sources of bias.
**Waterlander et al., 2013**		
Study design	Randomized controlled trails	
**Bias**	**Autors´ judgment**	**Support for judgement**
Random sequence generation (selection bias)	Low risk	Quote: "Individually randomly assigned by using a Random Number Generator in Microsoft Excel (Microsoft Corporation) into 1 of 4 study conditions."
Allocation concealment (selection bias)	Unclear risk	Not described in sufficient detail.
Blinding of participants and personnel (performance bias)	Low risk	Not clear if blinding but outcome not likely to be influenced by lack of blinding.
Blinding of outcome assessment (detection bias)	Low risk	Outcome was measured by raw sales data. Blinding of outcome assessment ensured.
Incomplete outcome data (attrition bias)	Low risk	Missing outcome data balanced in number across intervention groups, with similar seasons for missing data across groups.
Selective reporting (reporting bias)	Low risk	Study protocol available and all of the study´s pre-specified outcomes that are of interest have been reported in the pre-specified way.
Other bias	Low risk	Study appears to be free of other sources of bias.
**Winett et al., 1997**		
Study design	Randomized controlled trails	
**Bias**	**Autors´ judgment**	**Support for judgement**
Random sequence generation (selection bias)	Unclear risk	Insufficient detail about the sequence generation process. Unclear why some participants were control and others intervention.

Allocation concealment (selection bias)	Unclear risk	Not described in sufficient detail.
Blinding of participants and personnel (performance bias)	Low risk	Participants were likely to be blinded as they did not know that they were part of experiment. Cashiers received information about the goals of the study but were not able to distinguish between control and intervention participants based on the loyalty card.
Blinding of outcome assessment (detection bias)	Low risk	Outcome was measured by raw sales data. Blinding of outcome assessment ensured.
Incomplete outcome data (attrition bias)	Unclear risk	Insufficient reporting on if any participants dropped out.
Selective reporting (reporting bias)	Unclear risk	Insufficient information to assess whether an important risk of bias exists. Study protocol is not available.
Other bias	Unclear risk	No information on conflicting interests and the system is patent pending.
**Ernst et al., 1986**		
Study design	Controlled before and after studies	
**Bias**	**Autors´ judgment**	**Support for judgement**
Random sequence generation (selection bias)	High risk	Not a RCT.
Allocation concealment (selection bias)	High risk	Not a RCT.
Blinding of participants and personnel (performance bias)	Low risk	Intervention and control sites were in two different cities. Unlikely that participants know that they were part of intervention. Personnel not blinded but unlikely to impact outcome. People applying intervention are not the same as those assessing the outcome.
Blinding of outcome assessment (detection bias)	High risk	Outcome was measured by self-reported purchase data. Blinding of outcome assessment not ensured.
Incomplete outcome data (attrition bias)	Low risk	All participants completed the study and there were no losses to after/post-study period, no intervention withdrawals, no changes made to intervention or control sites and no major adverse events
Selective reporting (reporting bias)	High risk	Report on only some outcome variables while intervention was on an all-healthy product. Does not show effect of tests on specific outcome measurements for sales data. One or more outcome of interest in the review are reported incompletely so that they cannot be entered in a metanalysis.
Other bias	Unclear risk	Insufficient information to access whether an importaint risk of bias exists.
**Gamburzew et al., 2016**		
Study design	Controlled before and after studies	
**Bias**	**Autors´ judgment**	**Support for judgement**
Random sequence generation (selection bias)	High risk	Not a RCT.
Allocation concealment (selection bias)	High risk	Not a RCT.
Blinding of participants and personnel (performance bias)	Low risk	Sales data collected both before and after collected retrospectively, which ensures blinding of outcome assessment.
Blinding of outcome assessment (detection bias)	Low risk	Outcome was measured by raw sales data. Blinding of outcome assessment ensured.
Incomplete outcome data (attrition bias)	Low risk	All participants completed the study and there were no losses to after/post-study period, no intervention

		withdrawals, no changes made to intervention or control sites and no major adverse events
Selective reporting (reporting bias)	Unclear risk	Insufficient information to assess whether an important risk of bias exists. Study protocol is not available. No clear answer for why certain outcome measurements were chosen. One or more outcome of interest in the review are reported incompletely so that they cannot be entered in a metanalysis.
Other bias	Unclear risk	Study states that the 2 intervention and 2 control sites were simular in size, nr. of employees, costumer size. However no specific characteristics were described in the study which makes it unclear if baseline of control and intervention stores are comparable.
**Hobin et al., 2017**		
Study design	Controlled before and after studies	
**Bias**	**Autors´ judgment**	**Support for judgement**
Random sequence generation (selection bias)	High risk	Not a RCT.
Allocation concealment (selection bias)	High risk	Not a RCT.
Blinding of participants and personnel (performance bias)	Low risk	Intervention and control sites were in two different cities. Unlikely that participants know that they were part of intervention. Personnel not blinded but unlikely to impact outcome. People applying intervention are not the same as those assessing the outcome.
Blinding of outcome assessment (detection bias)	Low risk	Sales data collected both before and after collected retrospectively, which ensures blinding of outcome assessment.
Incomplete outcome data (attrition bias)	Low risk	All participants completed the study and there were no losses to after/post-study period, no intervention withdrawals, no changes made to intervention or control sites and no major adverse events
Selective reporting (reporting bias)	Unclear risk	Insufficient information to assess whether an important risk of bias exists. Study protocol is not available. No clear answer for why certain outcome measurements were chosen. One or more outcome of interest in the review are reported incompletely so that they cannot be entered in a metanalysis
Other bias	Low risk	Study showes that significant differences in participants characteristics in control and interventin sites exist (eg. In terms of age, gender, education). However sites are simular in terms of % overweight participants. Education level may have impact on effect of intervention but we have insufficent information to access whether these differences indroduce bias.
**Jeffery et al., 1982**		
Study design	Controlled before and after studies	
**Bias**	**Autors´ judgment**	**Support for judgement**
Random sequence generation (selection bias)	High risk	Not a RCT.
Allocation concealment (selection bias)	High risk	Not a RCT.
Blinding of participants and personnel (performance bias)	Low risk	Unlikely that participants know that they were part of intervention. Personnel not blinded but unlikely to impact outcome. People applying intervention are not the same as those assessing the outcome.

Blinding of outcome assessment (detection bias)	Low risk	Sales data collected both before and after collected retrospectively, which ensures blinding of outcome assessment.
Incomplete outcome data (attrition bias)	Low risk	All participants completed the study and there were no losses to after/post-study period, no intervention withdrawals, no changes made to intervention or control sites and no major adverse events
Selective reporting (reporting bias)	Unclear risk	Insufficient information to assess whether an important risk of bias exists. Study protocol is not available. No clear answer for why certain outcome measurements were chosen. One or more outcome of interest in the review are reported incompletely so that they cannot be entered in a metanalysis. No protocol.
Other bias	Low risk	Control and intervention sites seam to be comparable at baseline as they are simular in characteristics (e.g. same city, same chain, education level of costumers, type of costumers.
**Kiesel & Villas-Boas, 2013**		
Study design	Controlled before and after studies	
**Bias**	**Autors´ judgment**	**Support for judgement**
Random sequence generation (selection bias)	High risk	Not a RCT.
Allocation concealment (selection bias)	High risk	Not a RCT.
Blinding of participants and personnel (performance bias)	Low risk	Participants not informed that they are part of a study. Personnel not blinded but unlikely to impact outcome. People applying intervention are not the same as those assessing the outcome.
Blinding of outcome assessment (detection bias)	High risk	Outcome was measured by self-reported purchase data. Blinding of outcome assessment not ensured.
Incomplete outcome data (attrition bias)	Low risk	All participants completed the study and there were no losses to after/post-study period, no intervention withdrawals, no changes made to intervention or control sites and no major adverse events
Selective reporting (reporting bias)	Unclear risk	Insufficient information to assess whether an important risk of bias exists. Study protocol is not available. No clear answer for why certain outcome measurements were chosen. One or more outcome of interest in the review are reported incompletely so that they cannot be entered in a metanalysis. No protocol.
Other bias	Unclear risk	Author gives information about characteristics of the different control and intervention sites. Treament and control sites seam simular in terms of population size, income level, houshold size and racial and ethnic composition. However, the number of treament stores is much lower than control stores which may lead to larger differences between to groups at baseline.
**Rahkovsky et al., 2013**		
Study design	Controlled before and after studies	
**Bias**	**Autors´ judgment**	**Support for judgement**
Random sequence generation (selection bias)	High risk	Not a RCT.
Allocation concealment (selection bias)	High risk	Not a RCT.

Blinding of participants and personnel (performance bias)	Low risk	Intervention and control sites were in two different cities. Unlikely that participants know that they were part of intervention. Personnel not blinded but unlikely to impact outcome. People applying intervention are not the same as those assessing the outcome.
Blinding of outcome assessment (detection bias)	Low risk	Nilsen database sales data collected both before and after collected retrospectively, which ensures blinding of outcome assessment.
Incomplete outcome data (attrition bias)	Low risk	All participants completed the study and there were no losses to after/post-study period, no intervention withdrawals, no changes made to intervention or control sites and no major adverse events
Selective reporting (reporting bias)	Unclear risk	Insufficient information to assess whether an important risk of bias exists. Study protocol is not available. No clear answer for why certain outcome measurements were chosen. One or more outcome of interest in the review are reported incompletely so that they cannot be entered in a metanalysis. No protocol.
Other bias	Unclear risk	Differ homogeneity baseline and control store characteristics in terms of racial and ethnic composition of customers are noticeable between the two groups. However, may not be importains for whole grain products sales.
**Cawley et al. 2015**		
Study design	Interrupted time series	
**Bias**	**Autors´ judgment**	**Support for judgement**
Was the intervention independent of other changes?	Low risk	Study reports that no other changes were made in supermarkets (e.g., Marketing) at the same and that intervention was not influenced by other confounding variables/historic events.
Was the shape of the intervention effect pre-spefified?	Low risk	Introduction of the labeling system was predicted to increase sales of nutrition items and reduce sales of less nutritious items.
Was the intervention unlikely to affect data collection?	Low risk	Intervention itself was unlikely to affect data collection since data was collected through aggregate sales data before and after the labeling system was introduced.
Blinding of participants and personnel (performance bias)	Low risk	Intervention was a real intervention and participants and personnel unlikely to be influenced by knowledge of the intervention.
Blinding of outcome assessment (detection bias)	Low risk	Outcome measurement was objective sales data and thus unlikely to have been influences by knowledge of the intervention.
Were incomplete outcome data adequately addressed?	Low risk	Study excluded only sales data that " cannot be exclusively classified as pre-treatment or post-treatment.", which should be excluded. No data was missing to post-study period, no intervention withdrawals, no changes made to intervention or control sites and no major adverse events.
Are reports of the study free of suggestion of selective outcome reporting?	Low risk	All products in stores were included in analysis and outcome was measured for total food, foods categorized as less nutritious and nutritious. Report included all expected outcomes
Was the study free from other risks of bias?	Low risk	The study controlled for seasonal trends. No protocol available but still see low risk of other biases.
**Ejlerskov et al., 2018**		
Study design	Interrupted time series	
**Bias**	**Autors´ judgment**	**Support for judgement**

Was the intervention independent of other changes?	Unclear risk	Researchers state that they do not know in other events took place within store at the same time as intervention. Study however added comparison group and multiple supermarkets, reduced the potential impact of wider marketing trends and changes in consumer preferences. However, study did not include a non-intervention store as comparison.
Was the shape of the intervention effect pre-spefified?	Low risk	The directional effects of implementing of healthy check-out were predicted to reduce sales of unhealthy checkout snacks.
Was the intervention unlikely to affect data collection?	Low risk	Intervention itself was unlikely to affect data collection since data was collected through aggregate sales data before and after the labeling system was introduced.
Blinding of participants and personnel (performance bias)	Low risk	Intervention was a real intervention and participants and personnel unlikely to be influenced by knowledge of the intervention.
Blinding of outcome assessment (detection bias)	Low risk	Outcome measurement was objective sales data and thus unlikely to have been influences by knowledge of the intervention.
Were incomplete outcome data adequately addressed?	Low risk	No missing data, no loss to follow-up no intervention withdrawals, no changes made to intervention or control sites and no major adverse events
Are reports of the study free of suggestion of selective outcome reporting?	Low risk	Outcome measurement was chosen based on survey done at included supermarkets where they were asked about which foods that had a check-out. Report included all expected outcomes.
Was the study free from other risks of bias?	Low risk	The study appears to be free of other sources of bias and controlled for seasonal trends. Other biases accented for are market share, con-interventions, differential customer base. Research question was pre-specified in protocol.

Elshiewy et al., 2018

Study design	Interrupted time series	
**Bias**	**Autors´ judgment**	**Support for judgement**
Was the intervention independent of other changes?	Unclear risk	Researchers do not specify if they accented for other events that might have taken place at the same time as baseline or intervention phase.
Was the shape of the intervention effect pre-spefified?	Low risk	Introduction of labeling system was predicted to lead to healthier sales.
Was the intervention unlikely to affect data collection?	Low risk	Intervention itself was unlikely to affect data collection since data was collected through panel sales data and intervention was a labeling system.
Blinding of participants and personnel (performance bias)	Low risk	Intervention was a real intervention and participants and personnel unlikely to be influenced by knowledge of the intervention.
Blinding of outcome assessment (detection bias)	Low risk	Outcome measurement was objective sales data and thus unlikely to have been influences by knowledge of the intervention.
Were incomplete outcome data adequately addressed?	Low risk	No missing data, no loss to follow-up no intervention withdrawals, no changes made to intervention or control sites and no major adverse events
Are reports of the study free of suggestion of selective outcome reporting?	Low risk	No indication that other outcomes would be of interest.
Was the study free from other risks of bias?	Low risk	The study appears to be free of other sources of bias and controlled for seasonal trends.

Holmes et al., 2012

Study design	Interrupted time series	
**Bias**	**Autors´ judgment**	**Support for judgement**
Was the intervention independent of other changes?	Unclear risk	Researchers do not specify if they accented for other events that might have taken place at the same time as baseline or intervention phase.
Was the shape of the intervention effect pre-spefified?	Low risk	Healthy food campaign was hypothesized to increase consumer purchasing behaviors of healthier foods
Was the intervention unlikely to affect data collection?	Low risk	The data was collected from routine sources before and after introduction of the healthy food campaign.
Blinding of participants and personnel (performance bias)	Low risk	Intervention was a real intervention and participants and personnel unlikely to be influenced by knowledge of the intervention.
Blinding of outcome assessment (detection bias)	Low risk	Outcome measurement was objective sales data and thus unlikely to have been influences by knowledge of the intervention.
Were incomplete outcome data adequately addressed?	Low risk	No missing data, no loss to follow-up no intervention withdrawals, no changes made to intervention or control sites and no major adverse events
Are reports of the study free of suggestion of selective outcome reporting?	Unclear risk	Does not specific why outcome was measured for the selected product categories. No protocol available.
Was the study free from other risks of bias?	Unclear risk	Does not specify if accounted for seasonal variations. Pre-intervention was in the spring and post-intervention in the fall, and study should therefore have controlled for seasonality.

Patterson et al., 1992

Study design	Interrupted time series	
**Bias**	**Autors´ judgment**	**Support for judgement**
Was the intervention independent of other changes?	Unclear risk	Researchers do not specify if they accented for other events that might have taken place at the same time as baseline or intervention phase. They however accented for price, store, and seasonal effects.
Was the shape of the intervention effect pre-spefified?	Low risk	Introduction of the general media campaign was predicted to improve sales.
Was the intervention unlikely to affect data collection?	Low risk	The data was collected from routine sources before and after introduction of the intervention.
Blinding of participants and personnel (performance bias)	Low risk	Intervention was a real intervention and participants and personnel unlikely to be influenced by knowledge of the intervention.
Blinding of outcome assessment (detection bias)	Low risk	Outcome measurement was objective sales data and thus unlikely to have been influences by knowledge of the intervention.
Were incomplete outcome data adequately addressed?	Low risk	No missing data, no loss to follow-up no intervention withdrawals, no changes made to intervention or control sites and no major adverse events.
Are reports of the study free of suggestion of selective outcome reporting?	Unclear risk	Does not specific why outcome was measured for the selected product categories. No protocol available.
Was the study free from other risks of bias?	High risk	Articles states that large differences exist in baseline between control and intervention stores (e.g.,

		socioeconomic differences). Analysis was confounded by lack of comparability between the two groups. Seasonality was addressed although the analysis.
**Sacks et al., 2009**		
Study design	Interrupted time series	
**Bias**	**Autors´ judgment**	**Support for judgement**
Was the intervention independent of other changes?	Low risk	Researchers do not specify if they accented for the fact that some products were promoted during intervention period.
Was the shape of the intervention effect pre-spefified?	Low risk	Introduction of a traffic-light label is predicted to help customers make healthier food choices.
Was the intervention unlikely to affect data collection?	Low risk	The data was collected from routine sources before and after introduction of the intervention.
Blinding of participants and personnel (performance bias)	Low risk	Intervention was a real intervention and participants and personnel unlikely to be influenced by knowledge of the intervention.
Blinding of outcome assessment (detection bias)	Low risk	Outcome measurement was objective sales data and thus unlikely to have been influences by knowledge of the intervention.
Were incomplete outcome data adequately addressed?	Low risk	No missing data, no loss to follow-up no intervention withdrawals, no changes made to intervention or control sites and no major adverse events.
Are reports of the study free of suggestion of selective outcome reporting?	Low risk	No indication that other outcomes would be of interest.
Was the study free from other risks of bias?	Low risk	The study appears to be free of other sources of bias and controlled for seasonal trends.
**Sigurdsson et al., 2011**		
Study design	Interrupted time series	
**Bias**	**Autors´ judgment**	**Support for judgement**
Was the intervention independent of other changes?	Unclear risk	Researchers do not specify if they accented for other events that might have taken place at the same time as baseline or intervention phase. They however accented for fluctuations regarding time of the day, number of costumers, day of the week.
Was the shape of the intervention effect pre-spefified?	Low risk	Introduction of better placement of healthy products is predicted to help consumers to choose healthy food products.
Was the intervention unlikely to affect data collection?	Low risk	The data was collected from routine sources before and after introduction of the intervention.
Blinding of participants and personnel (performance bias)	Low risk	Participants did not know that they were part of experiment and personnel unlikely to be influenced by knowledge of the intervention.
Blinding of outcome assessment (detection bias)	Low risk	Outcome measurement was objective sales data and thus unlikely to have been influences by knowledge of the intervention. People applying intervention are not the same as those assessing the outcome.
Were incomplete outcome data adequately addressed?	Low risk	No missing data, no loss to follow-up no intervention withdrawals, no changes made to intervention or control sites and no major adverse events.

Are reports of the study free of suggestion of selective outcome reporting?	Low risk	No indication that other outcomes would be of interest.
Was the study free from other risks of bias?	Unclear risk	Does not specify if accounted for seasonal variations. Study does not specify then pre- and post-intervention phases took place, only referred to as periods (1 period = 4 days).
**Walmsley et al., 2018**		
Study design	Interrupted time series	
**Bias**	**Autors´ judgment**	**Support for judgement**
Was the intervention independent of other changes?	High risk	Study states that other changes were made at the same time as intervention (placement of fruits and vegetables at the front of store and entrance-facing display of fruits and vegetables were replaced with chiller cabinets). Research do not describe in detail what the other "changes" were only that stores were renovated. therefore, likely that other changes than the intervention may have impacted sales.
Was the shape of the intervention effect pre-spefified?	Low risk	Authors specified which shape they expected intervention to take.
Was the intervention unlikely to affect data collection?	Low risk	The data was collected from routine and retrospectively before and after introduction of the intervention.
Blinding of participants and personnel (performance bias)	Low risk	Participants did not know that they were part of experiment and personnel unlikely to be influenced by knowledge of the intervention.
Blinding of outcome assessment (detection bias)	Low risk	Outcome measurement was objective sales data and thus unlikely to have been influences by knowledge of the intervention. People applying intervention are not the same as those assessing the outcome.
Were incomplete outcome data adequately addressed?	Low risk	No missing data, no loss to follow-up no intervention withdrawals, no changes made to intervention or control sites and no major adverse events
Are reports of the study free of suggestion of selective outcome reporting?	Low risk	No indication that other outcomes would be of interest.
Was the study free from other risks of bias?	Low risk	The study appears to be free of other sources of bias and excluded sales data from holidays.
